# lncRNA profiling in early-stage chronic lymphocytic leukemia identifies transcriptional fingerprints with relevance in clinical outcome

**DOI:** 10.1038/bcj.2016.77

**Published:** 2016-09-09

**Authors:** D Ronchetti, M Manzoni, L Agnelli, C Vinci, S Fabris, G Cutrona, S Matis, M Colombo, S Galletti, E Taiana, A G Recchia, S Bossio, M Gentile, C Musolino, F Di Raimondo, A Grilli, S Bicciato, A Cortelezzi, P Tassone, F Morabito, M Ferrarini, A Neri

**Affiliations:** 1 Department of Oncology and Hemato-Oncology, University of Milan, Milan, Italy; 2 Hematology Unit, Fondazione IRCCS Ca' Granda, Ospedale Maggiore Policlinico, Milan, Italy; 3 Molecular Pathology Unit, IRCCS-A.O.U. San Martino-IST, Genoa, Italy; 4 Hematology Unit, Department of Onco-Hematology, A.O. of Cosenza, Cosenza, Italy; 5 Biotechnology Research Unit, Aprigliano, A.O./ASP of Cosenza, Cosenza, Italy; 6 School and Division of Hematology, University Hospital 'G. Martino', Messina, Italy; 7 Department of Biomedical Sciences, Division of Haematology, University of Catania and Ferrarotto Hospital, Catania, Italy; 8 Center for Genome Research Dept. of Life Sciences, University of Modena and Reggio Emilia, Modena, Italy; 9 Department of Experimental and Clinical Medicine, Magna Graecia University, Catanzaro, Italy; 10 Scientific Direction, IRCCS-A.O.U. San Martino-IST, Genoa, Italy

## Abstract

Long non-coding RNAs (lncRNAs) represent a novel class of functional RNA molecules with an important emerging role in cancer. To elucidate their potential pathogenetic role in chronic lymphocytic leukemia (CLL), a biologically and clinically heterogeneous neoplasia, we investigated lncRNAs expression in a prospective series of 217 early-stage Binet A CLL patients and 26 different subpopulations of normal B-cells, through a custom annotation pipeline of microarray data. Our study identified a 24-lncRNA-signature specifically deregulated in CLL compared with the normal B-cell counterpart. Importantly, this classifier was validated on an independent data set of CLL samples. Belonging to the lncRNA signature characterizing distinct molecular CLL subgroups, we identified lncRNAs recurrently associated with adverse prognostic markers, such as unmutated *IGHV* status, CD38 expression, 11q and 17p deletions, and *NOTCH1* mutations. In addition, correlation analyses predicted a putative lncRNAs interplay with genes and miRNAs expression. Finally, we generated a 2-lncRNA independent risk model, based on lnc-IRF2-3 and lnc-KIAA1755-4 expression, able to distinguish three different prognostic groups in our series of early-stage patients. Overall, our study provides an important resource for future studies on the functions of lncRNAs in CLL, and contributes to the discovery of novel molecular markers with clinical relevance associated with the disease.

## Introduction

Chronic lymphocytic leukemia (CLL) is a clinically heterogeneous disease.^1, 2^ Over the past two decades, several studies have shown a relationship between such heterogeneity and the presence of cellular and molecular markers, such as *IGHV* mutational status,^3^ cytogenetic abnormalities and gene mutations.^4, 5, 6, 7, 8, 9, 10, 11, 12, 13^ In recent years, great attention has been devoted to long non-coding RNA (lncRNA) molecules, a large class of transcripts that represent over half of the mammalian non-coding transcriptome. LncRNAs are involved in many biological processes, such as transcriptional gene regulation, maintenance of genomic integrity, genomic imprinting, cell differentiation or development.^14, 15^ Their deregulation promotes tumor formation, progression and metastasis in many types of cancer,^16^ including hematologic malignancies.^17, 18^ In addition, recent evidence has suggested and/or demonstrated an interplay of the various non-coding species, especially lncRNAs and miRNAs, in cancer development.^19^ The number of known human lncRNA transcripts is still evolving. To date, approximately 110 000 human lncRNAs are annotated in LNCipedia repository, with many loci generating multiple transcripts.^20^

The knowledge of the role of lncRNAs in CLL is virtually absent. Garding *et al.*^21^ provided insights into the epigenetic mechanisms regulating the expression of the DLEU1 and DLEU2 genes, mapped to the critical region at chromosomal band 13q14.3 which is deleted in over 50% of patients with CLL.^9, 22^ DLEU2 hosts the miR-15a/16-1 cluster, known for its crucial role in CLL pathogenesis.^23^ The only additional evidence of the involvement of lncRNAs in CLL suggest a role for NEAT1 and lincRNA-p21 as novel elements of the p53-dependent DNA damage response machinery.^24^

Herein, we investigated the lncRNA expression profiles in a large prospective series of Binet A CLL patients and in distinct populations of normal B-cells, isolated from tonsils and peripheral blood B-lymphocytes. Additionally, we searched for a putative significant correlation between lncRNAs and miRNAs at expression levels. Finally, lncRNA expression patterns were correlated with prognosis and prognostic markers to outline their possible role in CLL outcome.

## Patients and methods

### Patients

The study included 217 newly diagnosed CLL patients prospectively enrolled from several Italian institutions in an observational multicenter study (clinicaltrial.gov #NCT00917540), as previously described.^25^

Peripheral blood mononuclear cells from CLL patients and healthy donors, and B-cell populations from tonsils (i.e. naïve B-cells (N), marginal zone (MZ)-like, germinal center (GC), switched memory (M) B-cells), tonsillar and bone marrow plasma cells were obtained as previously described.^25^ Highly enriched CD19+ B-cells were characterized for *IGHV* mutational status, CD38 and ZAP70 expressions, *NOTCH1* mutations, and cytogenetic alterations, that is, deletion of chromosome 13q (del13q), 11q (del11q), 17p (del17p) and trisomy chromosome 12 (12+), as previously described.^12, 26, 27, 28, 29^

### Transcriptional analyses

The expression profiles of the 217 samples was generated using GeneChip Gene 1.0 ST Array (Affymetrix Inc., Santa Clara, CA, USA) as previously described,^30^ and data have been deposited in the NCBI Gene Expression Omnibus database (GSE46261). To detect lncRNA expression, we applied a custom pipeline able to re-annotate the probes included in the Gene 1.0 ST expression array according to the LNCipedia-v3.1 annotation database. MiRNA profiling of the 217 samples was generated using Agilent Human miRNA microarray (G4470B; Agilent Technologies, Santa Clara, CA, USA).^31^

Principal component analysis was performed by singular value decomposition of the considered data expression matrix using the *prcomp* function in the stats package, and the results visualized using the *plot3d* function in the rgl package for R software. Supervised analyses were performed using the Significant Analysis of Microarrays (SAM) software version 5.00^32^ using the web application provided in the shiny package for R software (https://github.com/MikeJSeo/SAM). The differentially expressed lncRNAs discriminating CLL and normal B-cell counterpart were identified using Prediction Analysis of Microarrays as previously described.^33^ Hierarchical agglomerative clustering of patients was performed adopting Pearson's correlation and average as distance and linkage methods, respectively. Functional characterization of lncRNAs was performed using the GSEA software v.2.2.1^34^ and the gene sets from Hallmarks, Kegg and Reactome collections. Ranking of coding genes from genes sets used the lncRNAs expression as a continuous phenotype and the Pearson's correlation as metric. Gene sets were considered significant if the false discovery rate was ⩽0.25 under 1000 permutations.

### Statistical analysis

The Cox proportional-hazards model in the Globaltest package for R was used to test the association between lncRNA expression levels, assumed as continuous variables, and progression-free survival (PFS) in terms of time-to-first-treatment as clinical outcome. *Globaltest* was run with 100 000 permutations on lncRNAs that varied mostly over the data set, namely those whose standard deviation of the expression exceeded the median plus one median absolute deviation of all the standard deviations. The list of significantly associated lncRNA to the PFS was reduced to derive a prognostic lncRNA signature: briefly, *k*-means clustering was applied to each lncRNA to define high- and low-expressing samples and then test the clinical outcome of the two groups. The survival distributions identified by this approach were tested using Kaplan–Meier estimator and log-rank test, and *P*-values were calculated according to the standard normal asymptotic distribution (*survdiff* function of the survival R package). The Benjamini–Hochberg method was applied for multiple testing corrections. Independence between lncRNA models and common CLL prognostic factors (*IGHV* mutational status, CD38, ZAP70, *NOTCH* mutation and unfavorable cytogenetic aberrations, namely del11q, del17p and 12+) was assessed using the multivariate Cox proportional-hazards regression procedure by the *coxph* function in the survival R package. Kendall Tau correlations and Wilcoxon rank-sum tests were applied using standard functions in R base package.

miRNA targets custom predictions were obtained for lncRNA sequences using RNA-22 version 2.0 prediction algorithm,^35^ which allows customizing input sequences and parameters (https://cm.jefferson.edu/rna22/Interactive/). The RNA-22 *perl* script was run on the combination of the 286 miRNA and 395 lncRNA FASTA sequences obtained from the correlation analysis (*P*<0.05 after multiple testing adjustment). The default sensitivity/specificity ratio of 1.032 was chosen, and provided a minimum seed size of seven bases with only one mismatch exception, a minimum number of 12 paired-up bases in heteroduplex with −12 kcal/mol maximum folding energy, and no more than one G-U wobble in the seed region.

## Results

### LncRNA expression profiles in CLL and in distinct subgroups of normal B-cells

The lncRNAs expression profiles of leukemic cells from a cohort of 217 patients with early stage, Binet A CLL were investigated (Supplementary Table S1)^25^ and compared with those of different types of normal B-cells. These samples included six peripheral B-cell (pBC), four GC, three N, two MZ, three M, four tonsillar plasma cells and four bone marrow plasma cells.

To detect lncRNA expression, we applied a custom pipeline able to re-annotate the probes included in the Gene 1.0 ST expression array according to the LNCipedia annotation database. Such a strategy allowed us to investigate the expression levels of 1852 well-annotated and specific human lncRNAs, as previously reported.^36^

**Figure 1:**
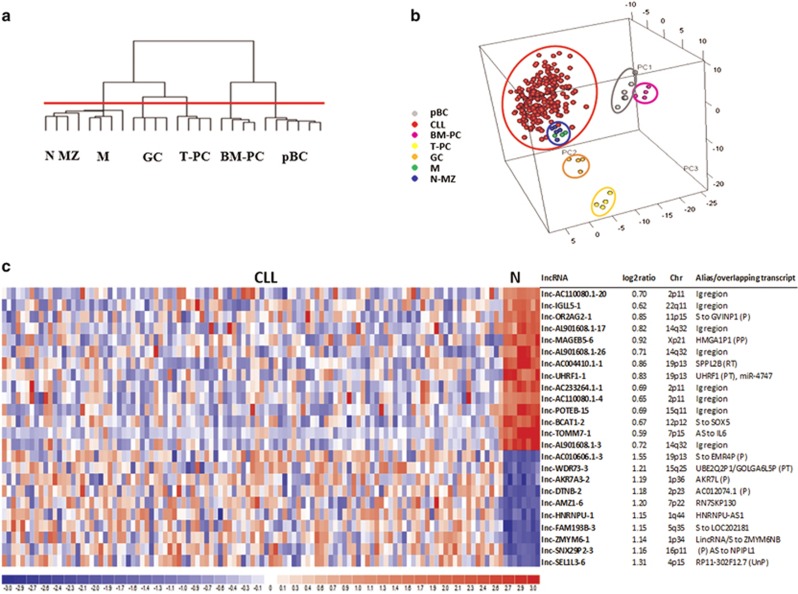
LncRNA expression profile of CLL and normal B-cells samples. (**a**) Hierarchical clustering of the 26 normal B-cell samples using the 141 lncRNAs most variable across the data set. The red line identified five branches related to the distinct normal B-cells subtypes. (**b**) Principal component analysis (PCA) that includes CLL samples shows that CLLs are closer in a three-dimensional space of similarity to M, N and MZ tonsillar B-lymphocytes than to other B-cell types, based on the expression of the 226 lncRNAs resulting as differentially expressed in the five normal B-cell subset from the multiclass analysis. (**c**) A heatmap showing the differentially expressed lncRNAs in the training group of CLL patients compared with normal (N) 'comparator' samples. For each lncRNA, information about chromosomal localization (Chr), alias name, transcripts overlapping in sense or antisense (indicated as S or AS, respectively) direction is indicated.

Firstly, we searched for the B-cell subset sharing lncRNA expression similarities with CLL cells. Clustering analysis of the 26 normal B-cell subtypes was run on the 141 lncRNAs varying at least 1.5-fold in expression levels from the mean across the data set. Distinct lncRNAs profiles were associated with tonsillar plasma cells, bone marrow plasma cells, pBCs and GC cells, whereas M, MZ and N B-cells presented undistinguishable expression profiles and were considered as part of a single cluster (Figure 1a). Among these five B-cells clusters, multiclass SAM analysis identified 226 differentially expressed lncRNAs (Supplementary Figure S1) that were subsequently used to compare normal B-cell subsets with CLL samples. The principal component analysis analysis revealed that CLL were more similar to tonsillar MZ, M and N B-cells than to GC cells, pBCs, tonsillar plasma cells or bone marrow plasma cells (Figure 1b). This finding is similar to that reported by our previous study in the context of miRNA and small nucleolar RNA (snoRNAs) expression in the same CLL data set.^25, 31^ Given the similarities of the lncRNA profile among CLL cells, tonsillar MZ, M and N B-cells as a whole were chosen as the 'comparator' B-cells to unravel lncRNA expression alterations in CLL. The classifier was first trained on an unbiased randomly selected cohort of 109 CLL cases (training set), and then applied to a validation set including the 108 remaining samples (Supplementary Table S1). The minimum error corresponded to 24 discriminant lncRNAs expression profiles between CLLs and comparator B-cells, leading to a good classification accuracy when applied to the validation patient set (overall: 99.14% specificity: 100% sensitivity: 99.07%) (Figure 1c). Specifically, the 14 lncRNAs that were downregulated in CLL included lnc-TOMM7-1 (showing the lowest expression ratio CLL/comparator) and 8 lncRNAs that are located in chromosomal regions, such as 14q32, 2p and 22q, coding for the highly variable portions of the immunoglobulin genes or for *IGHV* pseudogenes. The lncRNAs classifier included 10 lncRNAs that were upregulated in CLL samples, among which lnc-SNX29P2-3 and lnc-SEL1L3-6 (see below).

Importantly, the 24-lncRNA classifier signature was validated on a publicly available independent cohort including 9 CLL and 10 normal samples, profiled on the same array.^37^ Specifically, we verified the predictive power of the 24-lncRNA model using leave-one-out and linear discriminant analysis as the cross-validation procedure and prediction method, respectively, and demonstrated its capability to discriminate robustly CLL from normal B-cell samples (overall accuracy: 100%, Supplementary Figure S2).

### LncRNA expression in different CLL prognostic subgroups

CLL patients can be stratified into different prognostic groups based upon the presence/absence of cellular, molecular or cytogenetic markers. Here, we aimed at identifying specific lncRNAs signatures associated with each of these CLL subgroups (specified in Supplementary Table S1). We compared lncRNA expression of *IGHV* mutated (M-CLL, *n*=131) vs unmutated (UM-CLL, *n*=85) patients. The analysis identified 30 differentially expressed lncRNAs (Supplementary Table S2). To reduce biases possibly due to group heterogeneity (i.e., the distribution of cytogenetic alterations within the two *IGHV* classes), we restricted the analysis to a homogeneous CLL subgroup with del13q as common and sole abnormality, including 31 UM and 73 M-CLLs. This approach led to the identification of 12 differentially expressed lncRNAs (Table 1), 10 of which were already detected in the comparison of unselected M- and U-CLL (Supplementary Table S2). In particular, we identified an upregulation of the lnc-IRF2-3 mapping at 4q35 and lnc-AC004696.1-1, which is located antisense, head to head, to *ZNF667* gene at 19q13.

Overall, 12 and 20 lncRNAs distinguished CLL stratified based on ZAP70 or CD38 expression levels, respectively (Supplementary Tables S3 and S4). Among them, five lncRNAs (lnc-IRF2-3, lnc-AC004696.1-1, lnc-TNFRSF13B-5, lnc-C1orf132-1 and lnc-BACH1-1) were common to those identified in the above analysis (Supplementary Figure S3), which is in line with the notion that CD38 and ZAP70 represent in part surrogate markers of *IGHV* mutational status.

To identify lncRNA expression patterns characterizing the major cytogenetic aberrations, all the FISH 'negative' CLLs were considered as normal counterpart (78 patients). In addition, when del13q occurred with another cytogenetic abnormality, the latter was considered predominant for group inclusion. The results of each analysis are reported in Supplementary Tables S5–S8, while the most significant lncRNAs (top 10 score) are reported in Table 1. In detail, del13q patients showed an upregulation of lnc-SEL1L3-1, lnc-AMZ1-6, lnc-DTNB-2 and lnc-SNX29P2-3 (Supplementary Table S5), which were also downregulated in the comparator normal B-cells with respect to CLL samples (Figure 1c), and the downregulation of lnc-SPRYD7-1, also known as DLEU2. In addition, CLLs with del13q showed the downregulation of lnc-LIPG-3. This lncRNA has three different transcripts, including the independent transcriptional unit SCARNA17 and the non-coding RNA SNHG22, in the intron of which the SCARNA17 is located. Lnc-LIPG-3 is also downregulated in CLL samples with del11q and 12+, both of which showed an upregulation of lnc-AC004696.1-1. Only two lncRNAs were differentially expressed in patients with del17p. In particular, we found the downregulation of lnc-LTBP3-2 located at 11q13 antisense to both NEAT1 and MALAT1, the latter being a well-known putative oncogenic lncRNA found significantly correlated with lnc-LTBP3-2 expression in our CLL data set (*R*=0.54, *P*<2.2e-16).

Finally, we investigated the expression of lncRNAs specifically associated with the presence of *NOTCH1* mutations (Supplementary Table S9). Again, among the seven lncRNAs differentially expressed, *NOTCH1* mutated CLLs showed an upregulation of lnc-IRF2-3, lnc-AC004696.1-1 and lnc-BACH1-1, and a downregulation of lnc-C1orf132-1, all of which were found to be deregulated in CLL subgroups with adverse prognostic factors.

### LncRNAs interplay with genes and miRNAs in CLL

To gain evidence of lncRNAs that might influence gene expression, we evaluated the correlation between the expression level of the 1852 lncRNAs and that of the 17 788 coding transcripts unambiguously detected by the arrays. To be confident of predicting lncRNA–gene relationship, we focused on an lncRNAs/mRNAs ratio with a correlation coefficient >0.9 (Table 2), and found three distinct lncRNAs: (i) Lnc-AC004696.1-1 (see above) which positively correlated with its antisense overlapping gene *ZNF667*, suggesting a potential *in cis* regulation; (ii) lnc-NDST3-1, downregulated in del13q and del11q CLLs (Table 1), which positively correlated with the expression of *RPL18A*; and (iii) lnc-cYorf15A.1-2 that highly correlated with the expression of four genes located in sense or antisense orientation on chromosome Y about 7 Mb apart, and with two other genes, *RPS4Y1* and *ZFY*, located on the short arm of chromosome Y. Since lnc-cYorf15A.1-2 and its correlated genes are expressed by males only, the correlation coefficients were recalculated in male patients and found to be below 0.8 for all of the six lncRNA–gene couples.

The relationship between lncRNAs and miRNAs also was studied in all patients of the cohort.^31^ To test the transcriptional relationships, the correlations between the expression levels of any lncRNA and miRNA were computed first. We identified 861 lncRNA-miRNA couples significantly anti-correlated in our database (*q*-value<0.01). Then, one of the most common target prediction algorithms (RNA-22) was run on each significantly correlated pair, using the miRNA sequences annotated on miRbase v20 and the LNCipedia lncRNA sequences corresponding to the fragments investigated by the probes on the array. Hence, we identified 11 lncRNA-miRNA anti-correlated couples supported by miRNA target prediction (*q*-value<0.05; Table 3). Notably, we found the couples miR-331-3p/lnc-LIPG-3 (lnc-LIPG-3 downregulated in samples with del13q, del11q or 12+ Table 1), and miR-574-3p/lnc-LINS-1 (miR-574-3p reported as upregulated in UM-CLL patients).^31^

### Identification of an lncRNA transcriptional profile with clinical relevance

Since the cohort analyzed was recruited in a perspective study on Binet A patients, we could investigate correlations between lncRNA profiles and PFS. Clinical data were available for 209/217 CLL. To this aim, a *globaltest* was run on the 471 lncRNAs the expression of which varied mostly over the data set. Eight lncRNAs showed significant association with PFS (*P*<0.001; Supplementary Table S10) and five had a significant predictive value for PFS in univariate analysis (Table 4). The expression levels of lnc-IRF2-3, lnc-AC004696.1-1, lnc-C1orf132-1 and KIAA1755-4 were validated by qRT-PCR, showing a good concordance (Supplementary Figure S4 and Supplementary Table S11). Notably, lnc-KIAA1755-4 corresponds to the SNORA71A that is processed from the intronic region of the non-coding RNA SNHG17 (Supplementary Figure S4d).

**Figure 2:**
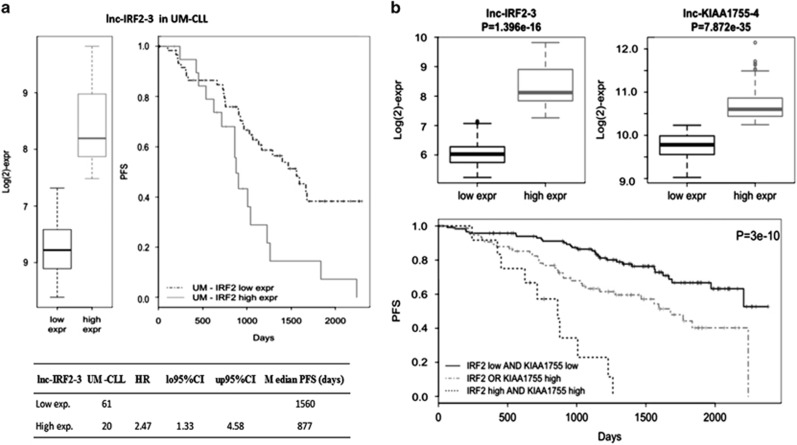
A 2-lncRNA risk model in CLL. (**a**) Progression-free survival of *IGHV* unmutated (UM)-CLL grouped according to lnc-IRF2-3 expression levels. Kaplan–Meier estimated curves of the two groups defined by lnc-IRF2-3 high (gray) and low (black) expression levels. (**b**) Kaplan–Meier estimated curves of the 2-lncRNA model. CLL are divided into a low-risk group (low expression of both lncRNAs), an intermediate group (high expression of one of the two lncRNAs) and a high-risk group (high expression of both lncRNAs). The high-risk group has a median PFS of 862 days.

Next, our analysis focused on lnc-IRF2-3 that had the highest predictive value for PFS by univariate test and was highly expressed in UM-CLLs. Interestingly, the highest lnc-IRF2-3 expression level was found in the subset of UM-CLL patients with the shortest PFS (Figure 2a). However, its predictive power lost strength (*P*=0.051) after adjustment for covariate confounders, particularly CD38, likely because high expression levels of CD38 and lnc-IRF2-3 define largely overlapping CLL subgroups (Supplementary Figure S3). In order to improve the robustness of the lncRNAs as PFS predictors, lnc-IRF2-3 was combined with a second lncRNA from the group with the significant predictive value (Table 4). We evaluated each of these lncRNAs in combination with lnc-IRF2-3, based on the following scheme defining groups as (i) high/high, (ii) low/low or (iii) discordant expression levels. Among all the possible combinations tested, the couple lnc-IRF2-3 and lnc-KIAA1755-4 determined the best predictive model. Specifically, a better PFS corresponded to patients with a concomitant low expression of both lncRNAs (a 'low-risk' group, including 122 patients, 56%), whereas a worse PFS was associated with their concomitant high expression ('high-risk' group, 12 patients; Figure 2b). The 'intermediate-risk' group (75 patients) was characterized by the discordant expression of the two lncRNAs. The high-risk group had a hazard ratio of 8.05 (95% CI: 3.82–16.96; median PFS: 862 days), and the intermediate-risk group of 2.27 (95% CI: 1.39–3.69; median PFS: 1678 days), compared with the low-risk group (median PFS not reached). Finally, multivariate regression analysis confirmed the independence of the 2-lncRNA risk model from other known predictive factors in CLL (*IGHV* mutational status, CD38 and ZAP70 expression, *NOTCH* mutation and unfavorable chromosomal aberration as covariates; Table 5). Likewise, the 2-lncRNA risk model resulted informative when compared with the recently defined progression-risk score (PRS) based on the integration of clinical, laboratory and biological parameters independently associated with PFS^38^ (Table 5).

**Figure 3:**
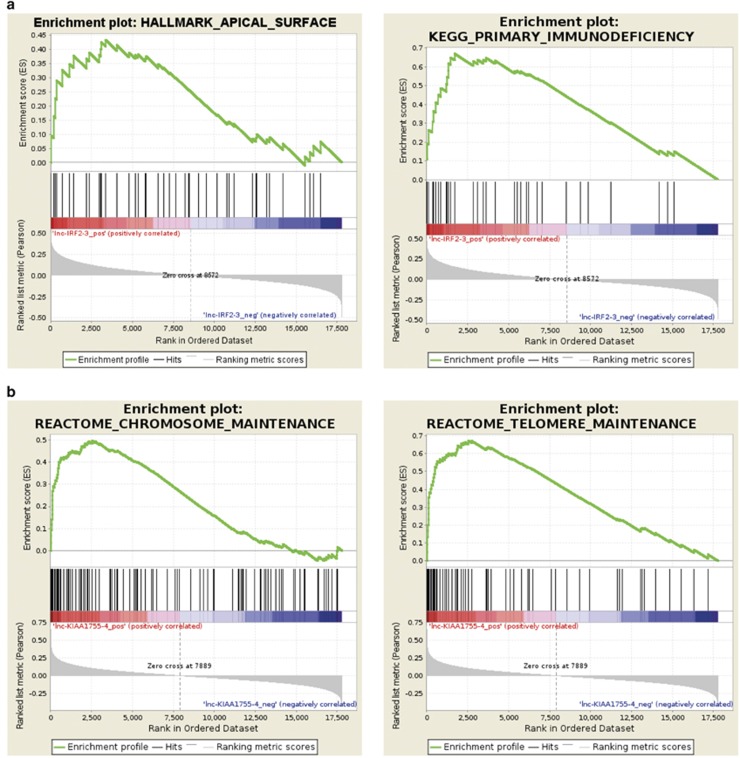
Representative enrichment plots of gene sets significantly up- and downregulated in CLL with increasing expression levels of lnc-IRF2-3 (**a**) or lnc-KIAA1755-4 (**b**) detected by gene set enrichment analysis. The green curves show the enrichment score and reflect the degree to which each gene (black vertical lines) is represented at the bottom of the ranked gene list.

To gain insight into their possible role in CLL pathophysiology, we evaluated *in silico* whether the modulation of lnc-IRF2-3 or lnc-KIAA1755-4 might correspond to transcriptional signatures possibly associated with functional categories. Specifically, we ranked genes according to decreasing Pearson's correlation with the expression level of lnc-IRF2-3 or lnc-KIAA1755-4 in the 217 samples using the gene set enrichment analysis software to identify *a priori*-defined sets of genes showing concordant modulation. We found lnc-IRF2-3 associated with 18 gene sets (1 from Kegg and 17 from Hallmarks data sets; Supplementary Table S12; representative examples in Figure 3a), virtually all related to the metabolism of amino acids, sugars and lipids. Interestingly, lnc-IRF2-3 resulted associated with the gene set linked to primary immunodeficiency, and with genes encoding proteins over-represented on the apical surface of epithelial cells (Figure 3a). Concerning lnc-KIAA1755-4, we identified 30 gene sets (6 from Kegg and 24 from Reactome data sets; Figure 3b and Supplementary Table S13), 10 of which including genes mainly encoding for ribosomal proteins, and thus principally associated with ribosome formation and translational processes. Notably, 12 gene sets highly enriched in genes encoding for histone components are associated with transcriptional processes, chromosomes and telomeres maintenance, and telomeres packaging.

## Discussion

This study provided a comprehensive analysis of the transcriptional profile of lncRNAs in a large cohort of early-stage CLL patients.

First, we unraveled a 24-lncRNA signature specifically deregulated in CLL compared with a normal B-cell counterpart. The identification of the normal counterpart is currently a debated issue.^37, 39^ Although based on a limited number of normal samples, our analysis revealed that the lncRNA expression profile of leukemic cells is more similar to N, MZ and M B-cells than to GC cells, total pBCs or PCs, as previously observed in our previous studies for miRNA and snoRNA expression patterns.^25, 31^ Therefore, lncRNAs that significantly discriminate CLL cells and N, MZ and M B-cells, may be of relevance in disease pathogenesis. Importantly, the 24-lncRNA model was validated on an independent data set. Among the 24 lncRNAs, we highlighted the downregulation of lnc-TOMM7-1, mapped to chromosome 7p antisense to the interleukin-6 (*IL6*) gene, which promotes B-cell lineage proliferation and differentiation. Lnc-TOMM7-1 may participate in IL6 transcriptional regulation and therefore may have a pathogenic role, given the potential function of IL6 as an autocrine growth factor in CLL.^40^ The lncRNAs classifier also included lnc-SNX29P2-3 and lnc-SEL1L3-6 which were upregulated in CLL samples. This finding, contradictory to that found by us in PC dyscrasia where the two lncRNAs are downregulated in pathological samples,^36^ suggests that the role of lncRNA likely depends on the cellular context.

Despite the homogeneity of the lncRNA expression profile, possibly enhanced by testing samples of patients uniformly at their early disease stages, specific lncRNA signatures were detected in subgroups of CLL stratified according to cellular, molecular and cytogenetic markers. As expected, some lncRNAs were recurrently associated with the presence of adverse prognostic markers. Among them, it is worth mentioning lnc-AC004696.1-1, also known as ZNF667-AS1, the highest expression of which was found in UM-CLLs as also showed by previous studies,^41^ and was also negatively associated with a short PFS. Interestingly, two recent studies have reported epigenetic silencing of this lncRNA during the immortalization of human mammary epithelial cells as well as in a panel of cancer types including acute myeloid leukemia, diffuse large B-cell lymphoma^42^ and solid tumors.^43^ These findings seem contradictory to the situation that we and others^41^ have found in CLL with adverse prognosis for which high lnc-AC004696.1-1 expression was compatible with a transformed and potentially aggressive condition, again supporting the notion that the role played by lncRNA likely depends on the cellular context. Notably, in our CLL database, lnc-AC004696.1-1 expression highly correlated with that of the *ZNF667* gene (Table 2), with which it shares (head to head) a CpG island. A co-regulatory mechanism of the two transcripts needs to be investigated, but it is of interest the evidence that ZNF667 inhibits the expression of the anti-apoptotic gene *BAX* in rats.^44^

Albeit it is feasible that lncRNAs are engaged in miRNA mediated interactions,^19^ their contribution is yet poorly explored in general, and in CLL in particular. To provide insights into this aspect, we studied the expression correlation of lncRNAs and miRNAs in paired samples, with the support of target prediction analysis. Among the 11 pairs reported in Table 3, particularly relevant appear miR-574-3p/lnc-LINS-1 and miR-331-3p/lnc-LIPG-3. MiR-574-3p is upregulated in UM-CLL and is associated with a major risk of disease progression in the same cohort of CLL.^31^ Considering that miR-574-3p has been described as a tumor suppressor miRNA in different types of solid tumors,^45^ we can hypothesize that in CLL, lnc-LINS-1 may play a fine-tuning role in the regulatory circuitry including miR-574-3p and its target genes. Considering the other miRNA/lncRNA pair, lnc-LIPG-3 expression was downregulated in samples with del13q, del11q or 12+ (Table 1). While existing data describe miR-331-3p as one of the most expressed miRNA in CLL as compared with normal peripheral CD19+ B-cells,^46^ our results do not highlight such a great difference with the normal comparators (Supplementary Figure S5) and is in all likelihood due to the different B-cell subpopulation chosen as normal controls. Notably, a network between miR-331-3p and lncRNAs has been already described in gastric cancer, where miR-331-3p has been proven to target the HOTAIR lncRNA that, in turn, functions as a competing endogenous RNA (ceRNA) to regulate *HER2* expression by sponging miR-331-3p.^47^

Finally, we have proposed a 2-lncRNA risk model, based on the expression of lnc-IRF2-3 and lnc-KIAA1755-4, able to stratify our series of early-stage Binet A CLL patients into three different prognostic groups. The model identifies a very high-risk group characterized by the concomitant high expression of both lncRNAs. Notably, the model is independent of the common prognostic markers and of a recently defined progression-risk score.^38^ Information on lnc-IRF2-3 is still very limited. Ferreira *et al.*^41^ reported its high expression in prognostically poor UM-patients (Supplementary Table S2). Conversely, lnc-IRF2-3 is progressively downregulated trough the more aggressive stages of PC dyscrasia.^36^ Such a discrepancy maybe due to the cellular context as previously hypothesized for lnc-SNX29P2-3 and lnc-SEL1L3-6. Our gene set enrichment analysis results pointed out that in CLL lnc-IRF2-3 expression is associated with 18 gene sets the majority of which (15) are related to the metabolism of amino acids, sugars and lipids. Among the remaining ones, it is of note the gene set linked to primary immunodeficiency given that it includes genes related to CLL biology such as Zap70, CD19, BTK and CD79A genes. In addition, we found a specific association between lnc-IRF2-3 expression and the gene set that included genes related the apical surface of epithelial cells, such as those important for determining cell polarity. Overall these data prompt to further investigation of the functional role of lnc-IRF2-3 in the biology and the progression of the disease. Lnc-KIAA1755-4 is processed from the intronic region of the non-coding RNA SNHG17 and actually corresponds to SNORA71A, thus belonging to a class of molecules essentially localized in the nucleolus where they function as guide RNAs for the post-transcriptional modification of ribosomal RNAs. In particular, SNORA71A is predicted to guide the pseudouridylation of U406 in 18S rRNA.^48^ The potential clinical relevance of this type of ncRNA family in cancer and CLL have been previously reported by us and others.^25, 49^ Functional analysis of genes whose expression might be related to lnc-KIAA1755-4 highlighted enrichments in genes encoding for histone components, genes associated with transcriptional processes, chromosome and telomere maintenance, and telomeres packaging, all of which are biological processes frequently affected during cellular immortalization and tumor progression.

Overall, our findings offer a portrait of the lncRNA transcriptional landscape in CLL, ultimately providing insights into the biological mechanisms involving the non-coding fraction of the transcriptome and contributing to suggest novel putative molecular markers associated with high-risk course of the disease.

## Figures and Tables

**Table 1:** LncRNAs resulting upregulated (↑) or downregulated (↓) from SAM analyses comparing CLLs with adverse and favorable prognostic factors

*lncRNA*^a^	*UM* *vs* *M*^b^	*del13* *vs* *Neg*^c^	*del11* *vs* *Neg*^c^	*12+* *vs* *Neg*^c^	*del17* *vs* *Neg*^c^	*Chr*	*Alias/overlapping transcript*^d^
**CPNE1-2**	↑	↑	↑			20q11	S to FER1L4 (P)
C20orf173-4	↑		↑			20q11	FER1L4 (P) (RI)
**AC004696.1-1**	↑		↑	↑		19q13	ZNF667-AS1
ACTR1B-5	↑		↑			2q11	S to ANKRD36B
IRF2-3	↑					4q35	Linc
BACH1-1	↑					21q21	LINC00189
FREM3-6	↑					4q31	AC097658.1 (P)
**CD38-2**	↑					4p15	TAPT1-AS1
**TNFRSF13B-5**	↑					17p11	TBC1D27 (UnP), TNFRSF13B (RI)
ZNF131-1	↑					5p12	LOC153684
**C1orf132-1**	↓					1q32	S to miR102 (miR-29B/29C)
KLHL9-1	↓					9p21	DKFZp686L0695
SNX29P2-3		↑	↑			16p11	AC009093.1 (P)
**GAS1-2**		↑^e^		↑		9q21	Linc
VKORC1L1-3		↑				7q11	RP13-254B10.1 (PP)
SEL1L3-6		↑				4p15	(UnP)
SPRYD7-1		↓				13q14	DLEU2
LIPG-3		↓	↓	↓		18q21	SNHG22 (SCARNA17)
ATG12-3		↓	↓			5q23	CTD-2287O16.1 (PP)
SYNCRIP-2		↓	↓			6q12	SNHG5
NDST3-1		↓	↓			4q26	SNHG8 (SNORA24)
LTBP3-2		↓^e^			↓	11q13	AS to NEAT1 and MALAT1
ATAD5-3		↓^e^			↓	17q11	SUZ12P1 (P)
ETNK2-4		↓				1q32	Linc
AC090699.1.1-1		↓^e^	↓			17q25	SNHG16
IQCG-11				↑		3q29	ANKRD18DP (P)
AC021860.1-2				↑		4p14	AS to KLF3
NANOGP1-1				↑		12p13	NANOGP1 (P)
SERPINB9-1				↑		6p25	S to miR-4645
JAM2-2				↓		21q21	MIR155HG
C9orf131-2				↓		9p13	RN7SL338P (M)
STYXL1-2				↓		7q11	TMEM120A (RI)

Abbreviations: PP, processed pseudogene; P, pseudogene; UnP, unprocessed pseudogene; M, miscellaneous RNA; RI, transcript with retained intron.

a lncRNAs in bold resulted associated with PFS.

b Analysis performed in the del13 CLL subgroup.

c FISH negative CLLs.

d Sense (S) to, or antisense (AS) to, overlapping transcripts; Ensembl type.

e Resulted from SAM analysis but not with the top 10 score.

**Table 2:** LncRNAs highly correlated with transcripts

*lncRNA*	*Transcript*	*lncRNA chr.*	*lncRNA alias*^a^	*Transcript chr.*	*Correlation coeff.*^b^
AC004696.1-1	ZNF667	19q13	ZNF667-AS1	19q13	0.94
NDST3-1	RPL18A	4q26	SNHG8	19p13	0.91
Cyorf15A.1-2	DDX3Y	Yq11	TXLNG2P	Yq11	0.97
Cyorf15A.1-2	KDM5D	Yq11	TXLNG2P	Yq11	0.96
Cyorf15A.1-2	USP9Y	Yq11	TXLNG2P	Yq11	0.98
Cyorf15A.1-2	UTY	Yq11	TXLNG2P	Yq11	0.92
Cyorf15A.1-2	RPS4Y1	Yq11	TXLNG2P	Yp11	0.96
Cyorf15A.1-2	ZFY	Yq11	TXLNG2P	Yp11	0.94

Chromosomal localization (Chr.) and alias name are indicated.

a Sense (S) to, or antisense (AS) to, overlapping transcripts.

b *P*-value=2.2e-16.

**Table 3:** LncRNAs highly correlated with miRNAs

*lncRNA*	*miRNA*	*lncRNA Chr.*	*lncRNA alias*^a^	*Correlation*		*Target prediction*	
				*cor*	q*-val*	*lncRNA target sequence from/to*	*q-val*
C1ORF86-1:2	*miR-221-3p*	1p36	AS to PRKCZ	−0.27	0.007	(668, 690)	0.011
MAP1LC3B2-2:4	*miR-30b-3p*	12q24	Linc00173	−0.27	0.007	(1890, 1911)	0.045
LIPG-3:2	*miR-331-3p*	18q21	SNHG22, SCARNA17	−0.26	0.008	(2485, 2506)	0.013
MON2-2:2	*miR-370-3p*	12q14	S to miR-let7i	−0.27	0.007	(10737, 10759)	0.019
TFDP2-7:3	*miR-486-3p*	3q23	GK5	−0.37	3.2E-05	(3303, 3321)	0.040
LINS-1:2	*miR-574-3p*	15q26	PRKXP1	−0.28	0.004	(5285, 5306)	<1E-06
DLK1-4:12	*miR-628-3p*	14q32	S to MEG3	−0.26	0.010	(4377, 4397)	0.043
C22ORF32-1:6	*miR-659-3p*	22q13	Linc-OGFRP1	−0.29	0.003	(2903, 2923)	0.023
SEC61G-12:1	*miR-659-3p*	7p12	RN7SKP218	−0.27	0.008	(98, 120)	0.047
KIDINS220-6:14	*miR-92a-1-5p*	2p25	Linc00298	−0.28	0.004	(184, 206)	0.011
SNHG15-1:8	*miR-940*	7p13	SNHG15	−0.35	1.0E-04	(2090, 2113)	0.049

Chromosomal localization (Chr.) and alias name are indicated.

a Sense (S) to, or antisense (AS) to, overlapping transcripts.

**Table 4:** LncRNAs with significant predictive value related to PFS in the univariate log-rank test

*lncRNA*	*Alias/overlapping transcript*	*Chr.*	*HR*	*LO 95% CI*	*UP 95% CI*	P*-value*^a^
IRF2-3	Linc	4q35	3.67	2.14	6.28	2.14E-06
C1orf132-1	S to miR-102	1q32	0.36	0.22	0.59	5.49E-05
ADAP2-2	RN7SL138P	17q11	0.37	0.22	0.63	0.000245
AC004696.1-1	ZNF667-AS1	19q13	2.67	1.69	4.22	2.29E-05
KIAA1755-4	SNORA71A	20q11	2.21	1.39	3.50	0.000691
ZNF131-1	Uncharacterized loc.	5p11	2.07	1.31	3.25	0.0016
PTPDC1-7	AS to MIRLET7D	9q22	0.45	0.28	0.74	0.00184
MTMR2-1	Pseudogene	11q21	0.53	0.33	0.84	0.00691

a Based on the K-means clustering stratification of CLL cases into two groups according to lncRNAs expression level, five out of the eight lncRNAs had a significant predictive value for PFS in the univariate log-rank test (*P*<0.001).

**Table 5:** Multivariate analysis comparing the 2-lncRNA risk model with prognostic variables or with PRS in CLL series

*Variable*	*HR*	*LO 95% CI*	*UP 95% CI*	P*-value*
I-RISK GROUP^a^	2.32	1.30	4.12	0.0043
H-RISK GROUP^a^	7.64	2.66	21.92	2E-04
UM-CLL	1.38	0.65	2.92	0.396
ZAP70+	0.84	0.43	1.60	0.596
del17+	1.67	0.54	5.11	0.366
del11+	3.98	1.72	9.19	0.001
12+	1.28	0.57	2.84	0.541
CD38+	1.00	0.99	1.01	0.243
*NOTCH*-MUT+	0.94	0.49	1.81	0.861
				
I-RISK GROUP^a^	1.54	0.85	2.78	0.149
H-RISK GROUP^a^	3.88	1.63	9.18	2E-03
PRS	3.18	2.08	4.83	6.5E-08

a Intermediate-risk or high-risk group in the 2 lncRNA-risk model.
