# Supplementary material for: lncRNA profiling in early-stage chronic lymphocytic leukemia identifies transcriptional fingerprints with relevance in clinical outcome
Source: Blood Cancer J. 2016 Sep 9;6(9):e468–. doi: 10.1038/bcj.2016.77 (PMC5056969; doi:10.1038/bcj.2016.77)
Supplement: Supplementary Information [file bcj201677x1.docx]

**Supplementary Information**

**[Supplementary Figures and Supplementary Tables.](file:///C:\\Users\\dome\\AppData\\Local\\Temp\\leu20165x1.doc#_Toc437959482)**

**Supplementary Figure S2.** Cross-validation of the 24-lncRNA signature in the independent database by Seifert *et al*., J. Exp. Med. 2012.

1200

800

400

0

**lnc-AC004696.1-1**

SAM

Score(d)

**6.8**

**9**

**20.7**

**CD38**

**ZAP70**

**UM-CLL**

**lnc-IRF2-3**

1000

800

600

400

200

0

**CD38**

**ZAP70**

**UM-CLL**

SAM

Score(d)

**4.9**

**5.3**

**6.8**

**lnc-C1orf132-1**

600

400

200

0

**CD38**

**ZAP70**

**UM-CLL**

SAM

Score(d)

**-4.5**

**-3.9**

**-8.8**

**Supplementary Figure S3.** Expression of lnc-AC004696.1-1, lnc-IRF2-3, and lnc-C1orf132-1 resulting from SAM analyses comparing CD38+ vs CD38-, ZAP70+ vs ZAP70-, and UM-CLL vs M-CLL in the CLL dataset. For all the analyses, the SAM score(d) of the respective lncRNA is reported on the right side of the panel. In each panel samples are ordered according to decreasing (lnc-AC004696.1-1, lnc-IRF2-3) or increasing (lnc-C1orf132-1) lncRNA expression levels. Under each patient, CD38, ZAP70, and UM status postivity are shown (+).

**Supplementary Figure S4.** Quantitative RT-PCR validation of lncRNA expression in 50 CLL patients. Genomic map of lnc-IRF2-3 (**a**), lnc-AC004696.1-1 (**b**), lnc-C1orf132-1 (**c**), and lnc-KIAA1755-4 (**d**). The scheme specified lncRNA transcripts (red) with custom primers (blue, sequences in Supplementary Table 13) used for SYBR Green qRT-PCR (10 ng of total RNA and SYBR™ Green master mixes). Pearson’s correlation coefficient was calculated between GEP data and quantitative RT-PCR results normalized using GAPDH gene expression and expressed as ΔCt.

**Supplementary Figure S5.** Hsa-miR-331-3p expression level in CLL and different types of normal B-cell purified from peripheral (BC) or tonsillar (T) samples (Negrini M *et al*. Clin.Cancer Res. 2014)

**Supplementary Table 1. Clinical, biological and molecular features of CLL samples.**

| **Feature** | | **N. of samples** | **Training set** | **Validation set** |
| --- | --- | --- | --- | --- |
| **Rai stage** | 0 | 162 | 87 | 75 |
|  | 1 | 40 | 15 | 25 |
|  | 2 | 15 | 7 | 8 |
| \| **IGVH** \| \| --- \| \|  \| \|  \| | M | 131 | 63 | 68 |
|  | UM | 85 | 45 | 40 |
|  | nd | 1 | 1 | 0 |
| **ZAP70** | Pos | 97 | 48 | 49 |
|  | Neg | 120 | 61 | 59 |
| **CD38** | Pos | 50 | 24 | 26 |
|  | Neg | 167 | 85 | 82 |
| **NOTCH** | Pos | 32 | 13 | 19 |
|  | Neg | 167 | 88 | 79 |
|  | nd | 18 | 8 | 10 |
| **FISH neg^a^** | yes | 78 | 39 | 39 |
|  | no | 138 | 69 | 69 |
|  | nd | 1 | 1 | 0 |
| **del 13q^b^** | yes | 104 | 52 | 52 |
|  | no | 112 | 56 | 56 |
|  | nd | 1 | 1 | 0 |
| **12+** | yes | 29 | 13 | 16 |
|  | no | 187 | 95 | 92 |
|  | nd | 1 | 1 | 0 |
| **del 11q** | yes | 16 | 10 | 6 |
|  | no | 200 | 98 | 102 |
|  | nd | 1 | 1 | 0 |
| **del 17p** | yes | 6 | 3 | 3 |
|  | no | 210 | 105 | 105 |
|  | nd | 1 | 1 | 0 |

^a^ FISH neg: samples with none of classical cytogenetic aberrations;

^b^ Biallelic 13q deletion was present in 18 samples.

**Supplementary Table 2. LncRNA resulting from SAM analysis comparing UM- vs M-CLLs.**

| **lncRNA** | **Score(d)** | **Fold Change** |
| --- | --- | --- |
| AC004696.1-1* | 20.69 | 11.63 |
| IRF2-3* | 6.82 | 1.77 |
| TNFRSF13B-5* | 5.51 | 1.55 |
| BACH1-1* | 5.04 | 1.39 |
| DEF8-2 | 4.30 | 1.22 |
| JMJD7-PLA2G4B-1 | 4.08 | 1.15 |
| JMJD7-PLA2G4B-2 | 4.08 | 1.15 |
| FREM3-6* | 4.08 | 1.16 |
| KB-1507C5.2.1-3 | 3.79 | 1.23 |
| SPRYD7-1 | 3.79 | 1.61 |
| AC010606.1-3 | 3.75 | 1.74 |
| C20orf173-4* | 3.54 | 1.42 |
| AC078802.1-2 | 3.53 | 1.27 |
| CD38-2* | 3.41 | 1.18 |
| ZNF583-4 | 3.40 | 1.32 |
| CPNE1-2* | 3.33 | 1.37 |
| ZNF816-2 | 3.28 | 1.24 |
| ACTR1B-5* | 3.18 | 1.39 |
| SLX1B-3 | 3.16 | 1.14 |
| ANKS4B-1 | 3.14 | 1.17 |
| TCL1B-3 | 3.10 | 1.28 |
| HSFY2-10 | 3.10 | 1.37 |
| ACSF3-1 | -3.22 | 0.87 |
| GTPBP4-1 | -3.48 | 0.85 |
| TAGLN-2 | -3.48 | 0.79 |
| CD46-4 | -3.66 | 0.89 |
| DPF3-2 | -3.78 | 0.87 |
| MTFP1-4 | -4.01 | 0.88 |
| MTMR2-1 | -4.13 | 0.79 |
| C1orf132-1* | -8.82 | 0.52 |
| ZNF131-1^#^ | 3.192 | 1.290 |
| KLHL9-1^#^ | -3.562 | 0.771 |

*lncRNAs differentially expressed in UM vs M-CLL also from the analysis in the CLL subgroup with del13 as the sole abnormality; # lncRNAs resulting only from the analysis in the del13 CLL subgroup.

**Supplementary Table 3. LncRNA resulting from SAM analysis comparing ZAP70+ vs ZAP70- CLLs.**

| lncRNA | Score(d) | Fold Change |
| --- | --- | --- |
| AC004696.1-1 | 9.004 | 4.847 |
| IRF2-3 | 5.351 | 1.578 |
| TNFRSF13B-5 | 4.076 | 1.384 |
| BACH1-1 | 3.439 | 1.257 |
| JMJD7-PLA2G4B-1 | 3.053 | 1.112 |
| JMJD7-PLA2G4B-2 | 3.053 | 1.112 |
| KB-1507C5.2.1-3 | 3.001 | 1.175 |
| ZBTB17-1 | 2.992 | 1.103 |
| DEF8-2 | 2.937 | 1.143 |
| FREM3-6 | 2.859 | 1.112 |
| C1orf132-1 | -3.905 | 0.723 |
| MTFP1-4 | -3.342 | 0.898 |

**Supplementary Table 4. LncRNA resulting from SAM analysis comparing CD38+ vs CD38- CLLs.**

| lncRNA | Score(d) | Fold Change |
| --- | --- | --- |
| AC004696.1-1 | 6.8822 | 4.4974 |
| IRF2-3 | 4.8859 | 1.6457 |
| TNFRSF13B-5 | 3.5676 | 1.4052 |
| SPRYD7-1 | 3.2213 | 1.6072 |
| HSFY2-10 | 3.0180 | 1.4281 |
| AC078802.1-2 | 2.9655 | 1.2638 |
| AC021860.1-2 | 2.9029 | 1.3559 |
| TYW5-4 | 2.8621 | 1.1612 |
| CD38-2 | 2.7369 | 1.1685 |
| CYorf17-2 | 2.7068 | 1.7280 |
| SERPINB9-1 | 2.6975 | 1.2070 |
| BACH1-1 | 2.5052 | 1.2228 |
| C1orf132-1 | -4.5357 | 0.6430 |
| MTMR2-1 | -3.4160 | 0.7909 |
| SLC7A1-1 | -2.9238 | 0.8550 |
| ACSF3-1 | -2.7955 | 0.8652 |
| KIF5B-1 | -2.7932 | 0.8886 |
| DTNB-2 | -2.7491 | 0.8393 |
| MTFP1-4 | -2.7218 | 0.9006 |
| C17orf46-3 | -2.6667 | 0.8513 |

**Supplementary Table 5. LncRNA resulting from SAM analysis comparing del13q vs FISH "negative" CLLs.**

| lncRNA | Score(d) | Fold Change | lncRNA | Score(d) | Fold Change |
| --- | --- | --- | --- | --- | --- |
| NDST3-1 | -6.204 | 0.540 | SNX29P2-3 | 5.076 | 1.331 |
| ATG12-3 | -5.088 | 0.804 | CPNE1-2 | 4.808 | 1.568 |
| LIPG-3 | -5.042 | 0.732 | VKORC1L1-3 | 4.644 | 1.186 |
| SYNCRIP-2 | -4.668 | 0.683 | SEL1L3-6 | 4.637 | 1.785 |
| SPRYD7-1 | -4.626 | 0.537 | OSBP2-2 | 4.504 | 1.192 |
| ETNK2-4 | -4.547 | 0.711 | MAGOHB-1 | 4.032 | 1.407 |
| FDXACB1-2 | -4.523 | 0.837 | DIRAS2-6 | 3.849 | 1.191 |
| RCOR3-1 | -4.449 | 0.825 | KLHL9-1 | 3.828 | 1.221 |
| GLG1-2 | -4.422 | 0.753 | AMZ1-6 | 3.781 | 1.243 |
| MDGA1-3 | -4.397 | 0.751 | ASNS-1 | 3.709 | 1.122 |
| METTL8-3 | -4.294 | 0.835 | RP11-210M15.2.1-2 | 3.540 | 1.120 |
| C9orf100-1 | -4.285 | 0.762 | POLR2J-1 | 3.514 | 1.119 |
| ANKRD36BP1-1 | -4.284 | 0.733 | GAS1-2 | 3.503 | 1.205 |
| NKX2-4-5 | -4.277 | 0.810 | DTNB-2 | 3.475 | 1.219 |
| MRPS7-6 | -4.267 | 0.850 | MAN2B1-1 | 3.455 | 1.160 |
| LTBP3-2 | -3.889 | 0.785 | ZMYM6-1 | 3.372 | 1.187 |
| ATAD5-3 | -3.851 | 0.775 | KANSL3-2 | 3.371 | 1.108 |
| SLC6A9-9 | -3.778 | 0.855 | EPHA7-8 | 3.370 | 1.134 |
| RSPH10B2-1 | -3.685 | 0.772 | AL901608.1-9 | 3.357 | 1.575 |
| FOXM1-4 | -3.551 | 0.807 |  |  |  |
| NDUFAF3-3 | -3.538 | 0.875 |  |  |  |
| MRPL40-5 | -3.508 | 0.853 |  |  |  |
| APC-6 | -3.470 | 0.825 |  |  |  |
| ARIH1-4 | -3.460 | 0.855 |  |  |  |
| TSN-3 | -3.365 | 0.767 |  |  |  |

**Supplementary Table 6. LncRNA resulting from SAM analysis comparing del11 vs FISH "negative" CLLs.**

| lncRNA | Score(d) | Fold Change |
| --- | --- | --- |
| CPNE1-2 | 5.599 | 2.599 |
| SNX29P2-3 | 5.328 | 1.634 |
| AC004696.1-1 | 5.072 | 7.110 |
| C20orf173-4 | 4.378 | 2.323 |
| ACTR1B-5 | 3.812 | 2.048 |
| NDST3-1 | -4.431 | 0.502 |
| AC090699.1.1-1 | -4.150 | 0.648 |
| ATG12-3 | -4.099 | 0.726 |
| SYNCRIP-2 | -3.448 | 0.597 |
| LIPG-3 | -3.438 | 0.703 |
| DPF3-3 | -3.258 | 0.783 |
| METTL8-3 | -3.249 | 0.782 |
| ARIH1-4 | -3.220 | 0.767 |
| HAUS5-1 | -3.194 | 0.659 |

**Supplementary Table 7. LncRNA resulting from SAM analysis comparing del17 vs FISH "negative" CLLs.**

| lncRNA | Score(d) | Fold Change |
| --- | --- | --- |
| ATAD5-3 | -3.221 | 0.598 |
| LTBP3-2 | -3.169 | 0.566 |

**Supplementary Table 8. LncRNA resulting from SAM analysis comparing 12+ vs FISH "negative" CLLs.**

| lncRNA | Score(d) | Fold Change |
| --- | --- | --- |
| LIPG-3 | -6.092 | 0.604 |
| STYXL1-2 | -4.456 | 0.743 |
| JAM2-2 | -4.181 | 0.567 |
| C9orf131-2 | -3.614 | 0.755 |
| XRN2-2 | -3.513 | 0.788 |
| CTC1-1 | -3.427 | 0.811 |
| KBTBD2-2 | -3.367 | 0.747 |
| WDR73-3 | -3.329 | 0.632 |
| APC-6 | -3.298 | 0.772 |
| METTL8-3 | -3.253 | 0.816 |
| AC021860.1-2 | 7.191 | 2.692 |
| SERPINB9-1 | 4.520 | 1.478 |
| IQCG-11 | 4.310 | 2.004 |
| GAS1-2 | 3.861 | 1.344 |
| AC004696.1-1 | 3.845 | 3.417 |
| NANOGP1-1 | 3.563 | 1.515 |
| RP11-210M15.2.1-2 | 3.434 | 1.177 |
| GAMT-2 | 3.378 | 1.184 |
| FOXP1-1 | 3.351 | 1.211 |
| DBX2-4 | 3.309 | 1.466 |
| MMADHC-1 | 3.237 | 1.291 |
| POLR2J-1 | 3.223 | 1.169 |

**Supplementary Table 9. LncRNA resulting from SAM analysis comparing Notch mutated vs wild-type CLLs.**

| lncRNA | Score(d) | Fold Change |
| --- | --- | --- |
| AC004696.1-1 | 5.128 | 3.947 |
| IRF2-3 | 4.986 | 1.812 |
| BACH1-1 | 3.028 | 1.315 |
| ANKRD30B-7 | 2.923 | 1.536 |
| C1orf132-1 | -3.845 | 0.646 |
| SQSTM1-1 | -3.224 | 0.740 |
| C8orf76-1 | -3.027 | 0.848 |

**Supplementary Table 10. List of lncRNAs significantly associated with Progression Free Survival** (**PFS).**

| **lncRNA** | **cytoband** | **Association with PFS** | **Global Test *P*-value** |
| --- | --- | --- | --- |
| IRF2-3 | 4q35 | - | 0.00001 |
| C1orf132-1 | 1q32.2 | + | 0.00002 |
| ADAP2-2 | 17q11.2 | + | 0.00003 |
| AC004696.1-1 | 19q13.43 | - | 0.00004 |
| PTPDC1-7 | 9q22.32 | + | 0.0002 |
| ZNF131-1 | 5p12 | - | 0.00057 |
| KIAA1755-4 | 20q11.23 | - | 0.00076 |
| MTMR2-1 | 11q21 | + | 0.00078 |
| CPNE1-2 | 20q11.22 | - | 0.00118 |
| AC010606.1-3 | 19p13.2 | - | 0.00127 |
| CD38-2 | 4p15.32 | - | 0.00127 |
| OR4E2-4 | 14q11.2 | - | 0.00129 |
| HNRNPU-1 | 1q44 | + | 0.0018 |
| TNFRSF13B-5 | 17p11.2 | - | 0.00185 |
| KLF14-1 | 7q32.3 | + | 0.00191 |
| TAMM41-3 | 3p25 | - | 0.00194 |
| MCL1-1 | 1q21.3 | + | 0.00228 |
| GALNT7-3 | 4q34 | - | 0.00243 |
| GPC5-1 | 13q31.3 | + | 0.00267 |
| BCAT1-2 | 12p12 | + | 0.0027 |
| PAPD4-3 | 5q14 | + | 0.00291 |
| MCL1-2 | 1q21.3 | + | 0.00318 |
| AL901608.1-18 | 14q32.33 | - | 0.00411 |
| ZNF701-1 | 19q13 | + | 0.00481 |
| STOM-7 | 9q33 | + | 0.00531 |
| GAS1-2 | 9q21.33 | - | 0.00549 |
| GCNT2-2 | 6p24.3 | - | 0.00608 |
| HSFY2-10 | Yq11 | - | 0.00736 |
| ANAPC7-1 | 12q24 | - | 0.00809 |
| ABLIM1-3 | 10q25 | + | 0.00932 |

LncRNAs are ordered according to the *p*-value obtained with the global test that measured the association (positive or negative, as indicated) between each lncRNAs and PFS. The 8 lncRNAs selected for further analysis (P<.001) were highlighted in grey.

**Supplementary Table 11. Sequence-specific SYBR green qRT-PCR primers**

| **Primer** | |
| --- | --- |
| Lnc-IRF2-3-F | GCAAAGGACCAAGAAAGCTG |
| Lnc-IRF2-3-R | CATACACAGGAGGCCTGGAT |
| Lnc-c1orf132-1-F | GGAACTGGCTCCTCTCCTCT |
| Lnc-c1orf132-1-R | CAAAGGCCCTACGAACACAT |
| Lnc-AC004696.1-1-F | AGTGTCCGCCATAAAGTCGT |
| Lnc-AC004696.1-1-R | GGGAATGAGTTGGTTGTTGG |
| Lnc-KIAA1755-4-F | CTCCTGCATCCGAAAGTGAT |
| Lnc-KIAA1755-4-R | ATAGGGTGGACCCTCCAAAC |

**Supplementary Table 12. Significant gene sets positively correlated to lnc-IRF2-3 expression (GSEA analysis).**

| **Geneset** | **NES** | ***P*-value** | **FDR** | **GENE SET SIZE** | **GENE LIST*** |
| --- | --- | --- | --- | --- | --- |
| HALLMARK_APICAL_SURFACE | 1.52 | 0.0227 | 2.27E-01 | 43 | **IL2RG, HSPB1, FLOT2, APP, AKAP7, MDGA1, NTNG1, NCOA6, DCBLD2, CX3CL1, TMEM8B, SULF2, SLC34A3**, BRCA1, LYPD3, IL2RB, SLC2A4, EFNA5, GSTM3, MAL, RTN4RL1, PKHD1, CROCC, AFAP1L2, SHROOM2, CD160, ATP6V0A4, RHCG, SLC22A12, ADAM10, GATA3, THY1, ATP8B1, EPHB4, PLAUR, B4GALT1, GAS1, LYN, PCSK9, AIM1, SCUBE1, SRPX, ADIPOR2 |
| KEGG_ALPHA_LINOLENIC_ACID_METABOLISM | 1.75 | 0.0061 | 1.52E-01 | 16 | **ACOX3, PLA2G1B, JMJD7-PLA2G4B, PLA2G4B, PLA2G4E, PLA2G2A, FADS2, PLA2G6**, PLA2G3, PLA2G2F, PLA2G2D, ACOX1, PLA2G12B, PLA2G10, PLA2G4A, PLA2G2E |
| KEGG_BETA_ALANINE_METABOLISM | 1.73 | 0.0108 | 1.58E-01 | 22 | **HIBCH, ALDH1B1, DPYD, SRM, ECHS1, GAD2, ABAT, ALDH3A2, ACADM**, ALDH7A1, HADHA, EHHADH, ALDH2, UPB1, AOC2, DPYS, ALDH9A1, MLYCD, GAD1, AOC3, CNDP1, SMS |
| KEGG_FRUCTOSE_AND_MANNOSE_METABOLISM | 1.77 | 0.0061 | 1.56E-01 | 33 | **PFKP, GMDS, PHPT1, MPI, ALDOC, TPI1, ALDOA, TSTA3, PFKL, FUK, PFKFB1, PFKM, GMPPA**, AKR1B1, PMM1, FPGT, FBP1, HK3, HK1, FBP2, ALDOB, KHK, MTMR2, PMM2, AKR1B10, HK2, MTMR1, GMPPB, PFKFB2, MTMR7, PFKFB4, PFKFB3, MTMR6 |
| KEGG_GALACTOSE_METABOLISM | 1.62 | 0.0163 | 2.10E-01 | 26 | **PFKP, GALK2, GANC, PFKL, GAA, B4GALT2, PFKM, GLB1, AKR1B1**, MGAM, HK3, HK1, GALE, UGP2, PGM1, GLA, GALK1, G6PC, HK2, GALT, LCT, B4GALT1, PGM2, LALBA, G6PC2, GCK |
| KEGG_GLYCEROLIPID_METABOLISM | 1.82 | 0.0000 | 2.40E-01 | 43 | **LPL, AGPAT4, DGKH, ALDH1B1, MBOAT1, LCLAT1, AKR1A1, AGPAT3, DGKA, AGPAT2, ALDH3A2**, AKR1B1, DAK, ALDH7A1, DGKD, DGKG, PNPLA3, ALDH2, DGAT2, LIPG, GLA, GK2, LIPC, LIPF, DGKZ, PPAP2A, ALDH9A1, GPAM, AGK, PNLIP, DGKE, PPAP2C, DGKQ, PPAP2B, CEL, MBOAT2, MGLL, DGKB, AWAT2, DGKI, AGPAT9, GLYCTK, AGPAT6 |
| KEGG_GLYCEROPHOSPHOLIPID_METABOLISM | 1.61 | 0.0122 | 2.09E-01 | 69 | **PLD1, AGPAT4, DGKH, MBOAT1, LCLAT1, PLA2G1B, LYPLA2, PLA2G15, JMJD7-PLA2G4B, PLA2G4B, AGPAT3, DGKA, AGPAT2, MBOAT7, GPD2, PLA2G4E, LPCAT4, PLA2G2A**, PLA2G6, PLD2, DGKD, ETNK2, PLA2G3, LPCAT3, PTDSS2, CHAT, DGKG, PLA2G2F, PGS1, PLA2G2D, LPGAT1, PHOSPHO1, PLA2G12B, GPD1L, LPCAT2, CDS1, DGKZ, CHKB, PPAP2A, TAZ, GPAM, PCYT1B, CDIPT, CHKA, DGKE, PPAP2C, PLA2G10, PCYT1A, PEMT, DGKQ, PPAP2B, PLA2G4A, MBOAT2, ETNK1, LPCAT1, GPD1, ACHE, PLA2G2E, DGKB, LCAT, PISD, DGKI, GNPAT, CRLS1, PTDSS1, AGPAT9, CHPT1, AGPAT6, CDS2 |
| KEGG_GLYCOLYSIS_GLUCONEOGENESIS | 1.77 | 0.0022 | 1.83E-01 | 56 | **PFKP, ALDH1B1, PCK2, GALM, AKR1A1, ALDOC, TPI1, ALDOA, ACSS2, GPI, PFKL, BPGM, PDHA1, ACSS1, ALDH3A2, PFKM, ALDH7A1, LDHB, GAPDH, ENO1**, FBP1, HK3, PGK2, HK1, ENO3, FBP2, ALDOB, ALDH2, PGM1, ALDH3A1, LDHA, ADH7, LDHAL6A, PGAM2, LDHC, G6PC, ADH4, ALDH9A1, HK2, ENO2, PKLR, PCK1, ADH1A, ADH6, PGM2, ALDH3B2, ADH1B, ADH5, G6PC2, ALDH1A3, DLAT, GCK, LDHAL6B, DLD, PDHA2, PDHB |
| KEGG_GLYCOSYLPHOSPHATIDYLINOSITOL_GPI_ANCHOR_BIOSYNTHESIS | 1.60 | 0.0287 | 2.07E-01 | 25 | **GPAA1, PIGV, PIGM, PIGF, PIGK, PIGU, PIGO, PIGS, PIGG, GPLD1, PIGL, PIGZ, PIGP**, PIGY, PIGX, PIGN, PIGT, PIGC, PIGQ, PIGA, DPM2, PGAP1, PIGH, PIGW, PIGB |
| KEGG_LIMONENE_AND_PINENE_DEGRADATION | 1.64 | 0.0194 | 1.91E-01 | 10 | **ALDH1B1, ECHS1, ALDH3A2, ALDH7A1, HADHA, NAT6, EHHADH**, ALDH2, YOD1, ALDH9A1 |
| KEGG_LINOLEIC_ACID_METABOLISM | 1.59 | 0.0267 | 2.10E-01 | 26 | **CYP2C8, PLA2G1B, JMJD7-PLA2G4B, PLA2G4B, CYP2J2, CYP3A43, PLA2G4E, PLA2G2A, PLA2G6**, PLA2G3, CYP2C9, PLA2G2F, PLA2G2D, PLA2G12B, AKR1B10, CYP2C18, CYP2E1, CYP3A7, CYP3A5, CYP1A2, PLA2G10, CYP2C19, ALOX15, PLA2G4A, CYP3A4, PLA2G2E |
| KEGG_PENTOSE_PHOSPHATE_PATHWAY | 1.72 | 0.0232 | 1.34E-01 | 26 | **PFKP, PRPS2, G6PD, TALDO1, H6PD, ALDOC, ALDOA, GPI, PFKL, RPIA, DERA, PFKM**, TKT, FBP1, PGD, FBP2, ALDOB, PGM1, TKTL2, TKTL1, PGLS, PRPS1L1, PGM2, RBKS, PRPS1, RPE |
| KEGG_PRIMARY_IMMUNODEFICIENCY | 1.99 | 0.0000 | 6.97E-02 | 33 | **ZAP70, IL2RG, BLNK, ICOS, BTK, CD19, TNFRSF13B, CD79A, IGLL1, ADA, CIITA, LCK, JAK3, CD40LG**, IKBKG, UNG, RFXAP, CD4, RFX5, CD8A, CD3D, IL7R, CD8B, DCLRE1C, RAG2, CD3E, RAG1, AICDA, RFXANK, AIRE, CD40, TNFRSF13C, PTPRC |
| KEGG_PROPANOATE_METABOLISM | 1.73 | 0.0243 | 1.41E-01 | 32 | **HIBCH, ALDH1B1, ECHS1, ACSS2, SUCLG2, ACSS1, ABAT, PCCA, ALDH3A2, ACACA, ACADM, ALDH7A1, LDHB, ALDH6A1, ACACB**, HADHA, EHHADH, MCEE, SUCLG1, ALDH2, LDHA, ACAT2, LDHAL6A, LDHC, PCCB, ALDH9A1, ACSS3, MLYCD, MUT, ACAT1, LDHAL6B, SUCLA2 |
| KEGG_PYRUVATE_METABOLISM | 1.78 | 0.0066 | 2.39E-01 | 39 | **ALDH1B1, PCK2, GLO1, MDH2, ACSS2, ACYP1, ME2, PDHA1, ACSS1, ME3, ALDH3A2, ACACA, HAGH, AKR1B1, ALDH7A1, LDHB, ACACB, ACOT12**, HAGHL, ALDH2, ME1, LDHA, LDHD, ACAT2, PC, GRHPR, LDHAL6A, LDHC, ALDH9A1, PKLR, PCK1, ACYP2, DLAT, ACAT1, LDHAL6B, DLD, MDH1, PDHA2, PDHB |
| KEGG_TRYPTOPHAN_METABOLISM | 1.55 | 0.0310 | 2.50E-01 | 37 | **ALDH1B1, KMO, ECHS1, OGDH, CAT, HAAO, ALDH3A2, GCDH, IL4I1, ALDH7A1, WARS, HADH, HADHA**, CYP1A1, AANAT, EHHADH, IDO2, MAOB, ALDH2, KYNU, ACMSD, OGDHL, INMT, ACAT2, AFMID, IDO1, AOX1, TDO2, TPH1, ALDH9A1, CYP1A2, MAOA, AADAT, DDC, ACAT1, TPH2, WARS2 |
| KEGG_VALINE_LEUCINE_AND_ISOLEUCINE_DEGRADATION | 1.72 | 0.0238 | 1.25E-01 | 42 | **HIBCH, ALDH1B1, ECHS1, BCAT1, IVD, MCCC1, BCAT2, AUH, BCKDHB, ABAT, ACAA2, PCCA, ALDH3A2, ACADM, IL4I1, BCKDHA, ALDH7A1, ALDH6A1, HADHB, HADH, HADHA**, HMGCL, EHHADH, MCEE, ALDH2, ACAA1, ACADS, ACAT2, HIBADH, AOX1, HMGCS2, PCCB, HMGCS1, ALDH9A1, ACAD8, HSD17B10, MUT, ACADSB, ACAT1, DLD, DBT, OXCT1 |
| KEGG_VIBRIO_CHOLERAE_INFECTION | 1.70 | 0.0105 | 1.28E-01 | 53 | **ATP6AP1, PDIA4, PRKCA, ACTB, ATP6V0A1, ADCY9, ACTG1, SEC61A1, ATP6V0E1, SEC61B, TCIRG1, KDELR2, PRKACA, KDELR1, PRKCB, ATP6V0B, ATP6V1G1**, ATP6V1C1, TJP2, ATP6V1C2, GNAS, MUC2, CFTR, ADCY3, ATP6V0E2, ERO1L, ATP6V1B1, PLCG1, ATP6V1E2, ATP6V1G3, PRKX, ATP6V0A4, ATP6V1B2, ARF1, KDELR3, PRKCG, PRKACB, SEC61A2, ATP6V0C, ATP6V1D, ATP6V0D2, KCNQ1, ATP6V1F, PRKACG, TJP1, SEC61G, ATP6V1A, ATP6V0A2, ATP6V1E1, ATP6V1H, PLCG2, SLC12A2, ATP6V0D1 |

* Genes contributing to the core enrichment in each gene set are indicated in bold.

**Supplementary Table 13. Significant gene sets positively correlated to lnc-KIAA1755-4 expression (GSEA analysis).**

| **Geneset** | **NES** | ***P*-value** | **FDR** | **GENE SET SIZE** | **GENE LIST *** |
| --- | --- | --- | --- | --- | --- |
| **REACTOME_TELOMERE_MAINTENANCE** | 2.07 | 0.0020 | 9.33E-03 | 66 | **HIST2H2AC, HIST1H4A, HIST1H2BB, HIST1H4C, HIST1H4F, HIST1H2AB, HIST3H3, HIST1H4B, HIST1H4L, HIST1H2AJ, RFC4, HIST1H2BI, HIST1H4D, HIST1H2BH, HIST1H2BO, HIST1H2AD, HIST1H2BE, HIST1H4H, HIST1H2BL, RPA2, TERF2, HIST1H2BM, HIST1H2BC, HIST1H2AE, HIST1H2BG, HIST1H4E, LIG1, HIST3H2BB, HIST4H4, FEN1, RPA3, POLE2, HIST1H2BN, PRIM1, HIST1H2BA, ACD, NHP2**, HIST1H2BF, RPA1, POLD2, RUVBL2, HIST1H2BJ, RFC2, TINF2, POT1, HIST1H2AC, DKC1, HIST1H2BD, TERT, HIST2H2BE, RFC5, POLD1, WRAP53, POLA2, DNA2, POLE, H2AFX, PCNA, RUVBL1, HIST1H2BK, HIST1H4I, TERF2IP, POLD3, RFC3, POLA1, TERF1 |
| **REACTOME_INFLUENZA_VIRAL_RNA_TRANSCRIPTION_AND_REPLICATION** | 2.08 | 0.0020 | 1.08E-02 | 65 | **RPLP2, RPS8, RPL18A, RPL12, RPSA, RPL35, RPS3, RPL29, RPL27, RPL31, RPL10A, RPL7A, POLR2L, RPL3, UBA52, RPL27A, RPS11, RPL11, RPS12, RPS19, POLR2J, RPL19, RPS6, RPS9, RPL5, RPL37, RPL23A, RPLP0, RPL22, RPS4X, POLR2G**, RPL15, RPS15, RPL38, RPL36, POLR2F, RPL34, RPL32, RPL26L1, HSP90AA1, RPL21, POLR2C, RPL8, POLR2E, RPS4Y1, RPL3L, RPL13, GTF2F2, POLR2D, RPS21, RPL35A, GRSF1, RPL37A, RPS3A, POLR2A, RPL14, RPL10, RPS23, GTF2F1, POLR2B, RPL28, RPL4, POLR2H, DNAJC3, IPO5 |
| **REACTOME_SRP_DEPENDENT_COTRANSLATIONAL_PROTEIN_TARGETING_TO_MEMBRANE** | 2.14 | 0.0000 | 1.28E-02 | 69 | **RPLP2, RPS8, RPL18A, RPL12, RPSA, RPL35, RPS3, RPL29, RPL27, RPL31, RPL10A, RPL7A, RPL3, UBA52, RPL27A, RPS11, RPL11, RPS12, DDOST, RPS19, TRAM1, RPL19, RPS6, RPS9, RPL5, RPL37, RPL23A, SEC11A, RPLP0, RPL22, RPS4X**, RPL15, RPS15, RPL38, SSR1, SPCS1, RPL36, SRP72, RPN2, RPL34, RPL32, RPL26L1, RPL21, RPL8, RPS4Y1, RPL3L, RPL13, SPCS3, RPN1, SEC61B, RPS21, SSR2, RPL35A, SSR3, SEC61A1, RPL37A, SEC61G, RPS3A, SEC61A2, RPL14, SRPRB, RPL10, RPS23, RPL28, SRPR, RPL4, SEC11C, SRP68, SRP54 |
| **REACTOME_AMYLOIDS** | 2.02 | 0.0080 | 1.54E-02 | 66 | **HIST2H2AC, HIST1H4A, HIST1H2BB, HIST1H4C, HIST1H4F, HIST1H2AB, HIST1H4B, HIST1H4L, HIST1H2AJ, HIST1H3F, HIST1H2BI, HIST1H3I, HIST1H4D, HIST1H3G, HIST1H3D, HIST1H2BH, HIST1H2BO, HIST1H3B, HIST1H2AD, HIST1H2BE, HIST1H4H, HIST1H2BL, HIST1H2BM, HIST1H3E, HIST1H2BC, HIST1H3C, HIST1H2AE, HIST1H2BG, HIST1H4E, HIST3H2BB, HIST4H4, HIST1H3J, HIST1H2BN, HIST1H2BA, APCS, HIST1H3H, NPPA, HIST1H3A, HIST1H2BF**, TGFBI, HIST1H2BJ, APP, IAPP, ODAM, GSN, ITM2B, HIST1H2AC, HIST1H2BD, LTF, HIST2H2BE, INS, SNCA, SAA1, FGA, PRL, MFGE8, HSPG2, H3F3B, CST3, H2AFX, SEMG1, HIST1H2BK, HIST1H4I, CALCA, LYZ, TTR |
| **REACTOME_PEPTIDE_CHAIN_ELONGATION** | 2.08 | 0.0000 | 1.58E-02 | 51 | **RPLP2, RPS8, RPL18A, RPL12, RPSA, RPL35, RPS3, RPL29, RPL27, RPL31, RPL10A, RPL7A, RPL3, UBA52, RPL27A, RPS11, RPL11, RPS12, RPS19, RPL19, RPS6, RPS9, RPL5, RPL37, RPL23A, RPLP0, EEF1A1, RPL22, RPS4X**, RPL15, RPS15, RPL38, RPL36, RPL34, RPL32, RPL26L1, RPL21, RPL8, RPS4Y1, EEF2, RPL3L, RPL13, RPS21, RPL35A, RPL37A, RPS3A, RPL14, RPL10, RPS23, RPL28, RPL4 |
| **REACTOME_PACKAGING_OF_TELOMERE_ENDS** | 1.94 | 0.0081 | 2.24E-02 | 41 | **HIST2H2AC, HIST1H4A, HIST1H2BB, HIST1H4C, HIST1H4F, HIST1H2AB, HIST3H3, HIST1H4B, HIST1H4L, HIST1H2AJ, HIST1H2BI, HIST1H4D, HIST1H2BH, HIST1H2BO, HIST1H2AD, HIST1H2BE, HIST1H4H, HIST1H2BL, TERF2, HIST1H2BM, HIST1H2BC, HIST1H2AE, HIST1H2BG, HIST1H4E, HIST3H2BB, HIST4H4, HIST1H2BN, HIST1H2BA, ACD, HIST1H2BF**, HIST1H2BJ, TINF2, POT1, HIST1H2AC, HIST1H2BD, HIST2H2BE, H2AFX, HIST1H2BK, HIST1H4I, TERF2IP, TERF1 |
| **REACTOME_NONSENSE_MEDIATED_DECAY_ENHANCED_BY_THE_EXON_JUNCTION_COMPLEX** | 1.94 | 0.0020 | 2.42E-02 | 72 | **RPLP2, RPS8, RPL18A, RPL12, RPSA, RPL35, RPS3, RPL29, RPL27, RPL31, RPL10A, RPL7A, RPL3, UBA52, RPL27A, RPS11, RPL11, RPS12, RPS19, RPL19, RPS6, RPS9, RPL5, RPL37, RPL23A, RPLP0, RPL22, RPS4X**, RPL15, RPS15, SMG8, RPL38, RPL36, RPL34, RPL32, RPL26L1, RPL21, RPL8, MAGOH, UPF3A, PPP2R1A, RPS4Y1, RPL3L, RPL13, GSPT2, SMG5, RPS21, RBM8A, RPL35A, RPL37A, EIF4G1, NCBP1, RPS3A, SMG7, ETF1, UPF3B, CASC3, RPL14, PPP2CA, RPL10, RPS23, RPL28, EIF4A3, UPF2, SMG9, RPL4, NCBP2, RNPS1, SMG6, PPP2R2A, SMG1, PABPC1 |
| **REACTOME_MEIOTIC_RECOMBINATION** | 1.95 | 0.0121 | 2.47E-02 | 70 | **HIST2H2AC, HIST1H4A, HIST1H2BB, HIST1H4C, HIST1H4F, HIST1H2AB, HIST3H3, HIST1H4B, HIST1H4L, HIST1H2AJ, HIST1H3F, HIST1H2BI, HIST1H3I, HIST1H4D, HIST1H3G, HIST1H3D, HIST1H2BH, HIST1H2BO, HIST1H3B, HIST1H2AD, HIST1H2BE, HIST1H4H, HIST1H2BL, RPA2, MND1, HIST1H2BM, HIST1H3E, HIST1H2BC, HIST1H3C, HIST1H2AE, HIST1H2BG, HIST1H4E, HIST3H2BB, HIST4H4, RPA3, HIST1H3J, HIST1H2BN**, HIST1H2BA, HIST1H3H, HIST1H3A, HIST1H2BF, RPA1, MSH4, ATM, HIST1H2BJ, BRCA1, RAD51C, HIST1H2AC, DMC1, HIST1H2BD, CDK2, HIST2H2BE, PRDM9, RAD50, BRCA2, H3F3B, CDK4, H2AFX, TEX15, HIST1H2BK, SPO11, HIST1H4I, TOP3A, NBN, RBBP8, MRE11A, BLM, MLH3, RAD51, MLH1 |
| **REACTOME_RNA_POL_I_PROMOTER_OPENING** | 1.96 | 0.0040 | 2.57E-02 | 48 | **HIST2H2AC, HIST1H4A, HIST1H2BB, HIST1H4C, HIST1H4F, HIST1H2AB, HIST1H4B, HIST1H4L, HIST1H2AJ, HIST1H3F, HIST1H2BI, HIST1H3I, HIST1H4D, HIST1H3G, HIST1H3D, HIST1H2BH, HIST1H2BO, HIST1H3B, HIST1H2AD, HIST1H2BE, HIST1H4H, HIST1H2BL, UBTF, HIST1H2BM, HIST1H3E, HIST1H2BC, HIST1H3C, HIST1H2AE, HIST1H2BG, HIST1H4E, HIST3H2BB, HIST4H4, HIST1H3J, HIST1H2BN, HIST1H2BA, HIST1H3H, HIST1H3A, HIST1H2BF**, HIST1H2BJ, MAPK3, HIST1H2AC, HIST1H2BD, HIST2H2BE, H3F3B, H2AFX, HIST1H2BK, HIST1H4I, MBD2 |
| **REACTOME_DEPOSITION_OF_NEW_CENPA_CONTAINING_NUCLEOSOMES_AT_THE_CENTROMERE** | 1.92 | 0.0061 | 2.57E-02 | 54 | **HIST2H2AC, HIST1H4A, HIST1H2BB, HIST1H4C, HIST1H4F, RSF1, HIST1H2AB, HIST1H4B, HIST1H4L, HIST1H2AJ, HIST1H2BI, HIST1H4D, HIST1H2BH, HIST1H2BO, HIST1H2AD, HIST1H2BE, HIST1H4H, HIST1H2BL, HIST1H2BM, HIST1H2BC, HIST1H2AE, HIST1H2BG, HIST1H4E, HIST3H2BB, HIST4H4, NPM1, HIST1H2BN, CENPH, HIST1H2BA**, HIST1H2BF, HIST1H2BJ, CENPP, HIST1H2AC, HIST1H2BD, CENPI, HIST2H2BE, CENPK, MIS18BP1, CENPQ, CASC5, ITGB3BP, H2AFX, MIS18A, OIP5, CENPO, RUVBL1, HIST1H2BK, HIST1H4I, HJURP, CENPA, SMARCA5, CENPN, RBBP7, RBBP4 |
| **REACTOME_TRANSLATION** | 1.97 | 0.0059 | 2.63E-02 | 101 | **RPLP2, RPS8, RPL18A, RPL12, RPSA, RPL35, RPS3, RPL29, EEF1B2, RPL27, RPL31, RPL10A, RPL7A, RPL3, UBA52, RPL27A, RPS11, EEF1G, RPL11, RPS12, EIF3E, DDOST, RPS19, TRAM1, RPL19, EIF3I, RPS6, EIF4A2, RPS9, RPL5, RPL37, EIF3G, RPL23A, SEC11A, RPLP0, EEF1A1, RPL22, RPS4X, EIF3K**, RPL15, RPS15, RPL38, SSR1, SPCS1, EIF4EBP1, RPL36, SRP72, RPN2, RPL34, RPL32, RPL26L1, RPL21, RPL8, RPS4Y1, EEF2, RPL3L, EIF2S2, RPL13, GSPT2, SPCS3, EIF2B1, RPN1, EIF3B, SEC61B, RPS21, SSR2, EIF5, EIF3H, RPL35A, EIF2S3, SSR3, SEC61A1, EIF2B2, RPL37A, EIF4G1, SEC61G, EIF4E, RPS3A, SEC61A2, ETF1, EIF2B5, EIF2B3, EIF2S1, RPL14, SRPRB, RPL10, RPS23, RPL28, SRPR, RPL4, EIF3D, SEC11C, EEF1D, EIF2B4, EIF5B, SRP68, EIF3A, EIF3F, EIF3J, SRP54, PABPC1 |
| **REACTOME_3_UTR_MEDIATED_TRANSLATIONAL_REGULATION** | 1.98 | 0.0020 | 2.75E-02 | 66 | **RPLP2, RPS8, RPL18A, RPL12, RPSA, RPL35, RPS3, RPL29, RPL27, RPL31, RPL10A, RPL7A, RPL3, UBA52, RPL27A, RPS11, RPL11, RPS12, EIF3E, RPS19, RPL19, EIF3I, RPS6, EIF4A2, RPS9, RPL5, RPL37, EIF3G, RPL23A, RPLP0, RPL22, RPS4X, EIF3K**, RPL15, RPS15, RPL38, RPL36, RPL34, RPL32, RPL26L1, RPL21, RPL8, RPS4Y1, RPL3L, EIF2S2, RPL13, EIF3B, RPS21, EIF3H, RPL35A, EIF2S3, RPL37A, EIF4G1, EIF4E, RPS3A, EIF2S1, RPL14, RPL10, RPS23, RPL28, RPL4, EIF3D, EIF3A, EIF3F, EIF3J, PABPC1 |
| **REACTOME_RNA_POL_I_TRANSCRIPTION** | 1.86 | 0.0177 | 4.26E-02 | 67 | **HIST2H2AC, HIST1H4A, HIST1H2BB, HIST1H4C, HIST1H4F, HIST1H2AB, HIST1H4B, HIST1H4L, HIST1H2AJ, HIST1H3F, HIST1H2BI, HIST1H3I, HIST1H4D, HIST1H3G, HIST1H3D, HIST1H2BH, HIST1H2BO, HIST1H3B, HIST1H2AD, HIST1H2BE, HIST1H4H, TAF1C, HIST1H2BL, PTRF, UBTF, HIST1H2BM, CBX3, HIST1H3E, HIST1H2BC, HIST1H3C, HIST1H2AE, POLR1D, HIST1H2BG, HIST1H4E, HIST3H2BB, HIST4H4, HIST1H3J, HIST1H2BN, GTF2H3, HIST1H2BA, HIST1H3H, HIST1H3A, HIST1H2BF**, HIST1H2BJ, ERCC2, MAPK3, HIST1H2AC, ERCC3, HIST1H2BD, HIST2H2BE, CCNH, POLR1C, H3F3B, H2AFX, HIST1H2BK, HIST1H4I, MBD2, GTF2H1, RRN3, KAT2B, POLR2H, TAF1B, MNAT1, POLR1B, ERCC6, TAF1A, POLR1A |
| **REACTOME_INFLUENZA_LIFE_CYCLE** | 1.87 | 0.0080 | 4.36E-02 | 96 | **RPLP2, RPS8, RPL18A, RPL12, RPSA, RPL35, RPS3, RPL29, RPL27, RPL31, RPL10A, RPL7A, POLR2L, RPL3, UBA52, RPL27A, RPS11, RPL11, RPS12, RPS19, POLR2J, RPL19, RPS6, NUP37, RPS9, RPL5, RPL37, RPL23A, NUP54, RPLP0, RPL22, RPS4X, POLR2G**, RPL15, RPS15, SEH1L, RPL38, RPL36, POLR2F, NUP62, RPL34, RPL32, RPL26L1, HSP90AA1, AAAS, NUP107, RPL21, POLR2C, RPL8, POLR2E, RAN, RPS4Y1, XPO1, RPL3L, NUP50, RPL13, GTF2F2, KPNB1, CALR, RAE1, POLR2D, RPS21, NUP85, NUP210, RPL35A, GRSF1, RPL37A, NUP43, CLTA, RPS3A, CLTC, TPR, NUP93, POLR2A, RPL14, RPL10, NUPL1, RPS23, GTF2F1, POLR2B, RPL28, NUP188, RPL4, NUP155, POLR2H, NUP205, NUP133, NUP153, NUP88, CANX, NUP35, RANBP2, DNAJC3, KPNA1, NUP214, IPO5 |
| **REACTOME_MEIOTIC_SYNAPSIS** | 1.84 | 0.0122 | 4.96E-02 | 63 | **HIST2H2AC, HIST1H4A, HIST1H2BB, HIST1H4C, HIST1H4F, HIST1H2AB, HIST3H3, HIST1H4B, HIST1H4L, HIST1H2AJ, HIST1H2BI, HIST1H4D, HIST1H2BH, HIST1H2BO, HIST1H2AD, STAG3, HIST1H2BE, HIST1H4H, HIST1H2BL, TERF2, HIST1H2BM, FKBP6, HIST1H2BC, HIST1H2AE, HIST1H2BG, HIST1H4E, HIST3H2BB, HIST4H4**, HIST1H2BN, HIST1H2BA, ACD, HIST1H2BF, UBE2I, HIST1H2BJ, BRCA1, SYCP1, TINF2, SUN2, REC8, POT1, TEX12, HIST1H2AC, HIST1H2BD, SYCP2, HIST2H2BE, LMNA, LMNB1, SMC1B, RAD21, H2AFX, HIST1H2BK, HIST1H4I, SYNE1, STAG2, TERF2IP, SYNE2, HSPA2, SMC3, ATR, STAG1, TERF1, DIDO1, SMC1A |
| **REACTOME_FORMATION_OF_THE_TERNARY_COMPLEX_AND_SUBSEQUENTLY_THE_43S_COMPLEX** | 1.78 | 0.0159 | 7.85E-02 | 27 | **RPS8, RPSA, RPS3, RPS11, RPS12, EIF3E, RPS19, EIF3I, RPS6, RPS9, EIF3G, RPS4X, EIF3K**, RPS15, RPS4Y1, EIF2S2, EIF3B, RPS21, EIF3H, EIF2S3, RPS3A, EIF2S1, RPS23, EIF3D, EIF3A, EIF3F, EIF3J |
| **REACTOME_CHROMOSOME_MAINTENANCE** | 1.78 | 0.0099 | 7.99E-02 | 107 | **HIST2H2AC, HIST1H4A, HIST1H2BB, HIST1H4C, HIST1H4F, RSF1, HIST1H2AB, HIST3H3, HIST1H4B, HIST1H4L, HIST1H2AJ, RFC4, HIST1H2BI, HIST1H4D, HIST1H2BH, HIST1H2BO, HIST1H2AD, STAG3, HIST1H2BE, HIST1H4H, HIST1H2BL, RPA2, TERF2, HIST1H2BM, FKBP6, HIST1H2BC, HIST1H2AE, HIST1H2BG, HIST1H4E, LIG1, HIST3H2BB, HIST4H4, FEN1, RPA3, NPM1, POLE2, HIST1H2BN, PRIM1, CENPH, HIST1H2BA, ACD, NHP2**, HIST1H2BF, UBE2I, RPA1, POLD2, RUVBL2, HIST1H2BJ, BRCA1, CENPP, SYCP1, RFC2, TINF2, SUN2, REC8, POT1, TEX12, HIST1H2AC, DKC1, HIST1H2BD, TERT, SYCP2, CENPI, HIST2H2BE, LMNA, RFC5, POLD1, LMNB1, WRAP53, CENPK, POLA2, MIS18BP1, CENPQ, CASC5, SMC1B, DNA2, RAD21, ITGB3BP, POLE, H2AFX, MIS18A, OIP5, CENPO, PCNA, RUVBL1, HIST1H2BK, HIST1H4I, SYNE1, STAG2, TERF2IP, SYNE2, HJURP, HSPA2, POLD3, CENPA, SMARCA5, CENPN, RFC3, POLA1, RBBP7, SMC3, ATR, STAG1, TERF1, DIDO1, SMC1A, RBBP4 |
| **REACTOME_MEIOSIS** | 1.79 | 0.0244 | 8.08E-02 | 97 | **HIST2H2AC, HIST1H4A, HIST1H2BB, HIST1H4C, HIST1H4F, HIST1H2AB, HIST3H3, HIST1H4B, HIST1H4L, HIST1H2AJ, HIST1H3F, HIST1H2BI, HIST1H3I, HIST1H4D, HIST1H3G, HIST1H3D, HIST1H2BH, HIST1H2BO, HIST1H3B, HIST1H2AD, STAG3, HIST1H2BE, HIST1H4H, HIST1H2BL, RPA2, TERF2, MND1, HIST1H2BM, HIST1H3E, FKBP6, HIST1H2BC, HIST1H3C, HIST1H2AE, HIST1H2BG, HIST1H4E, HIST3H2BB, HIST4H4, RPA3, HIST1H3J**, HIST1H2BN, HIST1H2BA, HIST1H3H, ACD, HIST1H3A, HIST1H2BF, UBE2I, RPA1, MSH4, ATM, HIST1H2BJ, BRCA1, RAD51C, SYCP1, TINF2, SUN2, REC8, POT1, TEX12, HIST1H2AC, DMC1, HIST1H2BD, CDK2, SYCP2, HIST2H2BE, LMNA, PRDM9, LMNB1, RAD50, BRCA2, H3F3B, SMC1B, RAD21, CDK4, H2AFX, TEX15, HIST1H2BK, SPO11, HIST1H4I, SYNE1, STAG2, TERF2IP, TOP3A, NBN, SYNE2, HSPA2, RBBP8, MRE11A, BLM, MLH3, RAD51, SMC3, ATR, STAG1, TERF1, DIDO1, SMC1A, MLH1 |
| **REACTOME_ENDOSOMAL_SORTING_COMPLEX_REQUIRED_FOR_TRANSPORT_ESCRT** | 1.77 | 0.0080 | 8.17E-02 | 24 | **UBA52, SNF8, CHMP5, VPS37D, VPS28, VPS36, VPS25, HGS, VPS4A, CHMP4B, TSG101**, VPS37A, CHMP4C, VPS37B, CHMP7, VTA1, CHMP2A, CHMP6, VPS37C, CHMP2B, STAM2, STAM, VPS4B, CHMP3 |
| **REACTOME_RNA_POL_I_RNA_POL_III_AND_MITOCHONDRIAL_TRANSCRIPTION** | 1.74 | 0.0319 | 1.06E-01 | 99 | **HIST2H2AC, HIST1H4A, HIST1H2BB, HIST1H4C, HIST1H4F, POLR2L, HIST1H2AB, HIST1H4B, HIST1H4L, HIST1H2AJ, HIST1H3F, HIST1H2BI, HIST1H3I, HIST1H4D, HIST1H3G, HIST1H3D, HIST1H2BH, HIST1H2BO, HIST1H3B, HIST1H2AD, HIST1H2BE, HIST1H4H, TAF1C, HIST1H2BL, PTRF, UBTF, HIST1H2BM, CBX3, HIST1H3E, SSB, HIST1H2BC, HIST1H3C, HIST1H2AE, POLR1D, HIST1H2BG, HIST1H4E, SNAPC2, HIST3H2BB, HIST4H4, HIST1H3J, LZTS1, HIST1H2BN, GTF3C5, GTF2H3, HIST1H2BA**, HIST1H3H, HIST1H3A, HIST1H2BF, POLR2F, HIST1H2BJ, GTF3C2, SNAPC4, SNAPC5, BRF2, ERCC2, POLR2E, POLRMT, NFIB, MAPK3, MTERF, HIST1H2AC, ERCC3, HIST1H2BD, POLR3GL, HIST2H2BE, SNAPC3, GTF3C3, CCNH, POLR3H, POLR1C, H3F3B, H2AFX, BRF1, HIST1H2BK, HIST1H4I, MBD2, POLR3D, GTF3C4, POLR3E, GTF2H1, POU2F1, RRN3, KAT2B, ZNF143, POLR3K, POLR2H, POLR3B, TAF1B, POLR3F, SNAPC1, POLR3A, TFB2M, TBP, MNAT1, POLR3C, POLR1B, ERCC6, TAF1A, POLR1A |
| **REACTOME_RESPIRATORY_ELECTRON_TRANSPORT_ATP_SYNTHESIS_BY_CHEMIOSMOTIC_COUPLING_AND_HEAT_PRODUCTION_BY_UNCOUPLING_PROTEINS_** | 1.69 | 0.0212 | 1.54E-01 | 66 | **COX6B1, NDUFA11, COX5A, CYC1, NDUFA7, COX7A2L, ATP5J, ATP5O, NDUFV1, NDUFB7, UQCRH, ATP5C1, NDUFA8, ATP5E, COX8A, NDUFA6, UCP1, NDUFB10, NDUFS7, NDUFS8, ETFA, NDUFS2, ATP5A1, NDUFB6, COX4I1, ETFB, CYCS**, NDUFS1, NDUFB9, ATP5G1, SDHC, NDUFB3, NDUFC2, NDUFA1, SDHB, ATP5B, ATP5J2, UQCRFS1, NDUFA12, COX6C, UQCRC1, NDUFA13, COX5B, NDUFA4, NDUFC1, NDUFV3, UCP2, NDUFS3, NDUFAB1, ATP5H, NDUFS6, UCP3, SDHD, UQCR11, NDUFB2, NDUFS5, UQCRQ, ETFDH, UQCRB, NDUFB8, SDHA, UQCRC2, NDUFA10, ATP5F1, NDUFA9, NDUFS4 |
| **REACTOME_ACTIVATION_OF_THE_MRNA_UPON_BINDING_OF_THE_CAP_BINDING_COMPLEX_AND_EIFS_AND_SUBSEQUENT_BINDING_TO_43S** | 1.68 | 0.0333 | 1.64E-01 | 32 | **RPS8, RPSA, RPS3, RPS11, RPS12, EIF3E, RPS19, EIF3I, RPS6, EIF4A2, RPS9, EIF3G, RPS4X, EIF3K**, RPS15, EIF4EBP1, RPS4Y1, EIF2S2, EIF3B, RPS21, EIF3H, EIF2S3, EIF4G1, EIF4E, RPS3A, EIF2S1, RPS23, EIF3D, EIF3A, EIF3F, EIF3J, PABPC1 |
| **REACTOME_BASE_FREE_SUGAR_PHOSPHATE_REMOVAL_VIA_THE_SINGLE_NUCLEOTIDE_REPLACEMENT_PATHWAY** | 1.62 | 0.0287 | 2.38E-01 | 10 | **SMUG1, NTHL1, MPG, MBD4, CCNO, MUTYH**, TDG, POLB, OGG1, APEX1 |
| **REACTOME_OXYGEN_DEPENDENT_PROLINE_HYDROXYLATION_OF_HYPOXIA_INDUCIBLE_FACTOR_ALPHA** | 1.62 | 0.0359 | 2.48E-01 | 14 | **TCEB2, UBA52, EGLN1, VHL, EGLN2**, UBE2D1, HIF1A, EGLN3, HIF3A, EPAS1, UBE2D3, CUL2, TCEB1, UBE2D2 |
| **KEGG_ALZHEIMERS_DISEASE** | 1.61 | 0.0060 | 2.12E-01 | 140 | **COX7A2, COX6B1, COX5A, CYC1, NDUFA4L2, NDUFA7, CALM3, COX7A2L, HSD17B10, ATP5J, ATP5O, NDUFV1, NDUFB7, UQCRH, ATP5C1, NDUFA8, BAD, ATP5E, COX8A, NDUFA6, FADD, NDUFB10, NDUFS7, NDUFS8, LPL, CAPN1, CACNA1F, PSEN2, NDUFS2, ATP5A1, NDUFB6, CDK5R1, ATP2A3, COX4I1, CYCS, CASP8, PPP3R2, PSENEN, CDK5**, TNFRSF1A, UQCR10, ATP5G2, NDUFS1, NDUFB9, PPP3R1, PLCB4, NOS1, ATP5G1, COX7B2, SDHC, NDUFB3, APP, PPP3CC, NDUFC2, NDUFA1, CAPN2, SDHB, PPP3CB, ATP5B, GRIN2C, GRIN2D, BACE1, BACE2, GAPDH, MAPK3, UQCRFS1, COX6C, UQCRC1, RYR3, GRIN1, APBB1, COX5B, NDUFA4, PLCB2, CASP7, CASP9, MME, FAS, ATP2A1, SNCA, CALML5, CACNA1C, CHP2, NDUFC1, COX8C, ATP5G3, CACNA1D, ITPR1, NAE1, GRIN2B, COX6B2, NDUFV3, CACNA1S, CALML3, MAPT, CALM1, NDUFS3, NDUFAB1, CASP3, BID, ATP5H, GRIN2A, CALML6, PPP3CA, ATP2A2, NDUFS6, COX4I2, LRP1, PSEN1, APAF1, SDHD, PLCB1, NCSTN, GNAQ, UQCR11, NDUFB2, EIF2AK3, NDUFS5, APOE, ITPR3, PLCB3, IDE, UQCRQ, ADAM10, ADAM17, GSK3B, UQCRB, NDUFB8, IL1B, SDHA, ITPR2, UQCRC2, ATF6, ERN1, NDUFA10, ATP5F1, CALM2, NDUFA9, NDUFS4, MAPK1 |
| **KEGG_GLYCOSAMINOGLYCAN_BIOSYNTHESIS_CHONDROITIN_SULFATE** | 1.71 | 0.0148 | 1.46E-01 | 22 | **CHPF2, CHST14, CHST12, B3GAT2, CHSY1, B3GAT3, CHST13, B4GALT7, B3GAT1, CHST7, CHPF, CHST3**, XYLT2, UST, CHSY3, CHST11, XYLT1, CHST15, DSE, B3GALT6, CSGALNACT2, CSGALNACT1 |
| **KEGG_OXIDATIVE_PHOSPHORYLATION** | 1.57 | 0.0240 | 2.28E-01 | 99 | **COX7A2, COX6B1, NDUFA11, COX5A, CYC1, NDUFA4L2, NDUFA7, COX7A2L, ATP5J, ATP5O, NDUFV1, NDUFB7, UQCRH, ATP5C1, PPA2, NDUFA8, ATP5E, ATP6V0B, COX8A, NDUFA6, NDUFB10, NDUFS7, ATP6V1G1, NDUFS8**, NDUFS2, ATP5A1, NDUFB6, COX4I1, ATP6V0E2, UQCR10, ATP6V1G3, ATP6V1E1, ATP5G2, NDUFS1, NDUFB9, LHPP, ATP5G1, COX7B2, SDHC, NDUFB3, COX11, NDUFC2, NDUFA1, ATP6V1F, SDHB, TCIRG1, ATP5B, ATP5J2, UQCRFS1, COX6C, ATP6V0D2, UQCRC1, ATP6V0E1, COX5B, NDUFA4, ATP6V1B1, NDUFC1, COX8C, ATP5G3, ATP6V0C, COX6B2, NDUFV3, ATP6V0D1, ATP6V1C2, ATP4A, NDUFS3, NDUFAB1, ATP5H, ATP12A, NDUFS6, PPA1, COX4I2, ATP6V1E2, ATP6V1C1, SDHD, COX15, ATP6V0A2, COX17, UQCR11, NDUFB2, NDUFS5, ATP6V1A, ATP6V0A4, UQCRQ, UQCRB, NDUFB8, ATP6AP1, SDHA, UQCRC2, ATP6V0A1, COX10, ATP4B, NDUFA10, ATP5F1, ATP6V1H, ATP6V1D, ATP6V1B2, NDUFA9, NDUFS4 |
| **KEGG_PARKINSONS_DISEASE** | 1.63 | 0.0201 | 2.16E-01 | 96 | **COX7A2, COX6B1, COX5A, CYC1, NDUFA4L2, NDUFA7, COX7A2L, ATP5J, ATP5O, NDUFV1, NDUFB7, UQCRH, ATP5C1, NDUFA8, ATP5E, COX8A, NDUFA6, PARK7, NDUFB10, NDUFS7, NDUFS8, HTRA2, SLC25A5, NDUFS2, ATP5A1, UBE2L6, NDUFB6, COX4I1, UCHL1, CYCS, LRRK2, SLC18A1, UQCR10, ATP5G2, NDUFS1, TH, NDUFB9**, UBB, ATP5G1, COX7B2, SDHC, NDUFB3, PINK1, NDUFC2, NDUFA1, SLC25A31, SDHB, ATP5B, UQCRFS1, COX6C, UQCRC1, GPR37, COX5B, UBA7, NDUFA4, SNCAIP, CASP9, SNCA, PARK2, UBE2G2, NDUFC1, COX8C, ATP5G3, COX6B2, UBA1, NDUFV3, NDUFS3, NDUFAB1, CASP3, ATP5H, SLC6A3, VDAC2, NDUFS6, COX4I2, APAF1, SDHD, UBE2J2, UBE2J1, UBE2G1, UQCR11, NDUFB2, NDUFS5, SLC18A2, UQCRQ, UQCRB, NDUFB8, SEPT5, SDHA, VDAC1, PPID, UQCRC2, NDUFA10, ATP5F1, SLC25A4, NDUFA9, NDUFS4 |
| **KEGG_RIBOSOME** | 2.17 | 0.0000 | 2.95E-03 | 55 | **RPLP2, RPS8, RPL18A, RPL12, RPSA, RPL35, RPS3, RPL29, RPL27, RPL31, RPL10A, RPL7A, RPL22L1, RPL3, UBA52, RPL27A, RPS11, RSL24D1, RPL11, RPS12, RPS19, RPL19, RPS6, RPL36AL, RPS9, RPL5, RPL37, RPL23A, RPLP0, RPL22, RPS4X**, RPL15, RPS15, RPL38, RPL36, RPL34, RPL32, RPL26L1, RPS27L, RPL21, RPL8, RPS4Y1, RPL3L, RPL13, MRPL13, RPS21, RPL35A, RPL37A, RPL10L, RPS3A, RPL14, RPL10, RPS23, RPL28, RPL4 |
| **KEGG_SYSTEMIC_LUPUS_ERYTHEMATOSUS** | 2.08 | 0.0080 | 4.51E-03 | 103 | **HIST2H2AC, HIST1H4A, HIST1H2BB, HIST1H4C, HIST1H2AI, HIST1H4F, HIST2H2AB, HIST1H2AB, HIST3H3, HIST1H4B, HIST1H4L, HIST1H2AJ, HIST1H2AK, HIST1H3F, HIST1H2BI, HIST1H3I, HIST1H4D, HIST1H3G, HIST1H3D, HIST1H2BH, HIST1H2BO, HIST1H3B, HIST1H2AD, HIST1H2BE, HIST1H4H, HIST1H2BL, HIST1H2AM, H2BFM, HIST1H2BM, HIST1H3E, SSB, HIST1H2BC, HIST1H3C, ACTN2, HIST3H2A, HIST1H2AE, HIST1H2AL, HIST1H2BG, H2AFY2, HIST1H2AH, HIST1H4E, HIST3H2BB, HIST4H4, HIST1H3J, TRIM21, HIST1H2BN, H2AFJ, HIST1H2BA, HIST1H3H**, HIST1H3A, HIST1H2BF, H2BFWT, C7, ELANE, HIST1H2BJ, SNRPB, FCGR2B, HIST1H2AG, HIST1H2AC, HLA-DQB1, HIST1H2BD, C1S, SNRPD3, HIST2H2BE, FCGR2A, TROVE2, FCGR3A, CTSG, CD80, HLA-DQA1, C8G, C8B, HLA-DRB1, C1R, GRIN2B, SNRPD1, C1QC, H3F3B, C5, CD28, C3, HIST1H4G, C8A, H2AFX, HIST1H2AA, GRIN2A, C1QB, HLA-DRB5, HIST1H2BK, HIST1H4I, H2AFV, IFNG, CD40, CD86, CD40LG, ACTN4, C6, C9, C1QA, H3F3C, ACTN1, H2AFY, IL10 |

*Genes contributing to the core enrichment in each gene set are indicated in bold.

**Supplementary Figure S1.** Cross-validation of the 24-lncRNA signature in the independent database by Seifert *et al*., J. Exp. Med. 2012.
